# Text messages to increase attendance to follow-up cervical cancer screening appointments among HPV-positive Tanzanian women (*Connected2Care*): study protocol for a randomised controlled trial

**DOI:** 10.1186/s13063-017-2215-x

**Published:** 2017-11-21

**Authors:** Ditte S. Linde, Marianne S. Andersen, Julius D. Mwaiselage, Rachel Manongi, Susanne K. Kjaer, Vibeke Rasch

**Affiliations:** 1 Department of Obstetrics and Gynaecology, Odense University Hospital, Odense, Denmark; 2 Institute of Clinical Research, University of Southern Denmark, Odense, Denmark; 3 OPEN, Odense Patient Data Explorative Network, Odense University Hospital, Odense, Denmark; 4 Department of Medical Endocrinology, Odense University Hospital, Odense, Denmark; 5 Department for Cancer Prevention Services, Ocean Road Cancer Institute, Dar es Salaam, Tanzania; 6 Institute of Public Health, Kilimanjaro Christian Medical University College, Moshi, Tanzania; 7 Department of Gynaecology, Rigshospitalet University Hospital, Copenhagen, Denmark; 8 Department of Virus, Lifestyle and Genes, Danish Cancer Society Research Center, Copenhagen, Denmark

**Keywords:** mHealth, SMS intervention, Mobile phone, HPV, Cervical cancer, Screening, RCT, Tanzania

## Abstract

**Background:**

Cervical cancer is a major health concern in Tanzania, caused by poor attendance for cervical cancer screening and follow-up of women at risk. Mobile telephone health interventions are proven effective tools to improve health behaviour in African countries. So far, no knowledge exists on how such interventions may perform in relation to cervical cancer screening in low-income settings. This study aims to assess the degree to which a Short Message Service (SMS) intervention can increase attendance at appointments among women who have tested positive for high-risk (HR) Human Papillomavirus (HPV) during cervical cancer screening.

**Methods/design:**

*Connected2Care* is a non-blinded, multicentre, parallel-group, randomised controlled trial. Tanzanian women testing positive to HR HPV at inclusion are randomly assigned in an allocation ratio of 1:1 to the SMS intervention or the control group (standard care). In a period of 10 months, the intervention group will receive 15 one-directional health educative text messages and SMS reminders for their appointment. The total sample size will be 700 with 350 women in each study arm. Primary outcome is attendance rate for follow-up. Secondary objectives are cost-effectiveness, measured through incremental ratios, and knowledge of cervical cancer by a 16-item true/false scale questionnaire at baseline and follow-up. Barriers against implementing the intervention will be assessed in a mixed-methods sub-population study.

**Discussion:**

This study may provide information on the potential effects, costs, and barriers in implementing an SMS intervention targeting a group of women who are followed up after testing positive for HR HPV and are, therefore, at increased risk of developing cervical cancer. This can guide decision-makers on the effective use of mobile technology in a low-income setting. Trial status: recruiting.

**Trial registration:**

ClinicalTrials.gov, ID: NCT02509702. Registered on 15 June 2015.

**Electronic supplementary material:**

The online version of this article (doi:10.1186/s13063-017-2215-x) contains supplementary material, which is available to authorized users.

## Background

Human Papillomavirus (HPV) is the most common sexually transmitted infection in the world and approximately 80% of all sexually active persons will be infected with HPV at some point during their lifetime [1, 2]. Persistent high-risk (HR) HPV infection is a necessary first step in developing cervical pre-cancerous lesions. A minority of these lesions progress to invasive cervical cancer over 10–20 years. The natural history of infection to cancer is complex, and many factors influence this, including the type of HPV, the number of sexual partners, parity, other sexually transmitted agents, and host susceptibility [3]. Cervical cancer often affects women of reproductive age and is a major health challenge in low-income countries (LICs) such as Tanzania where it accounts for 38% (*n* = 7300) of all female cancers, and 34% (*n* = 4200) of all female cancer-related deaths [1, 4]. Human immunodeficiency virus (HIV)-infected women are at high risk of contracting HPV infection and developing cervical cancer. Further, cervical cancer screening is limited in Tanzania and the screening uptake is poor due to lack of knowledge of the disease and its prevention. These factors are contributing to the large cervical cancer burden in Tanzania, and the disease is a public health concern with enormous social and economic impact [1–3, 5–10]. It is yet to be estimated to which degree women, who have attended screening and have had abnormal test results, will attend follow-up screening appointments in African populations.

As with many other African nations, Tanzania is skyrocketing into the mobile era. By the end of 2014, 75% of the population (34 million) had a mobile subscription [5]. The opportunity to give mobile technology a formal role in health care is increasingly being recognised [11]. The Ministry of Health in Tanzania has Mobile Health (mHealth) as part of its electronic health strategy from 2013 to 2018 [12]. Randomised controlled trials (RCTs) have documented that mobile technology can have a positive effect on both patients’ and health personnel’s behaviours in high- as well as in low-income settings [11, 13–20]. In East Africa, mHealth interventions have proven to increase attendance with skilled delivery staff among pregnant women (by 13%); adherence to anti-retroviral therapy among HIV-positive patients (by 12–13%); attendance at post-operative clinic visits among men who have been circumcised for HIV prevention (by 5.7%); and correct malaria-case management among health personnel (by 23–24%) [13–16, 20]. However, mHealth initiatives are still in their early days in LIC, and for full effect it is key to consider literacy, cultural, technical and scalability issues during implementation [11].

To our knowledge, no RCTs have previously tested the degree to which text messages can improve cervical cancer screening behaviour and follow-up of women who have had abnormal screening results. However, RCTs from high-income countries have shown that educative text messages and Short Message Service (SMS) reminders can significantly improve other types of cancer preventive behaviour; for example self-reported skin cancer prevention, breast self-examinations and breast cancer screening attendance [17–19].

The present article describes the study protocol for an RCT that aims to investigate the association between the SMS intervention *Connected2Care* and attendance at cervical cancer screening follow-up appointments among HPV-positive women in Tanzania. Based on the effect of other mHealth initiatives in LIC [14, 15, 21], the hypothesis is that health educative text messages and SMS reminders will increase by 15% the attendance rate to follow-up appointments among HPV-positive women. Additionally, in order to consider the real-world feasibility of implementing the intervention in a low-income setting, the study aims to investigate the cost-effectiveness of the intervention as well as barriers for implementation.

## Methods/design

### Study objectives

#### Primary objective

- To assess the effect of an SMS intervention on Tanzanian HPV-positive women’s attendance for cervical cancer screening follow-up appointments at 14 months compared to standard care

#### Secondary objectives

- To estimate the cost-effectiveness of an SMS intervention targeting Tanzanian HPV-positive women who have a 14-month follow-up screening appointment
- To assess the effect of an SMS intervention on Tanzanian HPV-positive women’s knowledge of cervical cancer and screening
- To understand barriers against the implementation of an SMS intervention in Tanzania in a mixed-methods sub-population study

### Setting

The study will be conducted at three health facilities in Tanzania—one in the Dar es Salaam Region and two in the Kilimanjaro Region. All sites will be urban or semi-urban areas. The study site in Dar es Salaam will be the cervical cancer screening clinic at Ocean Road Cancer Institute (ORCI). The two study sites in the Kilimanjaro Region will be the reproductive health clinics and the care and treatment clinics (CTCs) at (1) Kilimanjaro Christian Medical Centre (KCMC) and (2) Mawenzi Regional Referral Hospital in Moshi.

### Study design

#### Intervention

**Fig. 1:**
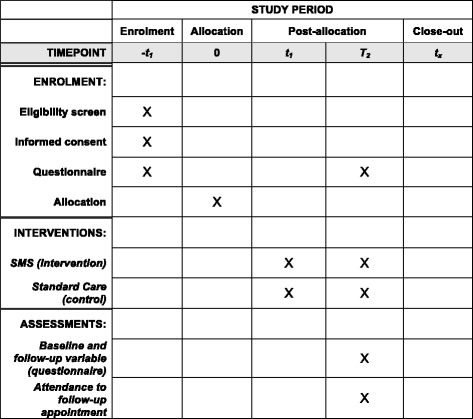
Standard Protocol Items: Recommendations for Interventional Trials (SPIRIT) Figure. Schedule of enrolment, intervention, and assessment

*Connected2Care* is a non-blinded, multicentre, parallel-group, randomised controlled trial. Tanzanian women testing positive to HR HPV at inclusion are randomly assigned in an allocation ratio of 1:1 to the SMS intervention or the control group (standard care). Once women testing positive for HPV have been informed of their test result, all eligible participants will be uploaded to the SMS system used for the intervention. The randomisation will occur through an incorporated algorithm in the SMS system and automatically assign 50% to a ‘no SMS group’ (control group) and 50% to an ‘SMS group’ (intervention group). The control group will receive standard care, which is a follow-up appointment at 14 months written on an appointment card. In addition to standard care, the intervention group will receive the SMS intervention (Fig. 1 Standard Protocol Items: Recommendations for Interventional Trials (SPIRIT) Figure. For the full SPIRIT Checklist see Additional file 1). The study is non-blinded due to the nature of the intervention, which will be overt for both the intervention group receiving the SMS and the control group not receiving any SMS. Health personnel who screen the women for cervical cancer will not be aware of the participants’ randomisation status until they attend follow-up.

The SMS intervention will consist of 15 text messages that will be sent to the intervention group over a period of 10 months. There will be two types of text messages: (1) educational text messages, and (2) SMS reminders for the follow-up appointment. The cervical cancer educational messages will be sent once a month and concern risk factors, common symptoms and screening information. The SMS reminders will inform the women of their appointment date and encourage women who have missed their follow-up appointment to attend the screening (Table 1). The reminders will be sent 2 weeks, 1 week and 1 day prior to the appointment date, as well as 1 day and 1 week after the appointment. The messages are phrased in a polite, caring, educational and encouraging manner. The messages were developed in English and translated to Kiswahili in a standard forward with backward translation. The maximum number of characters in each message is 480 as this is equivalent to three text messages. This is the maximum number of messages that basic mobile phones can string together. This means that each health educative text message or SMS reminder will be received as *one* message on all mobile phones.

**Table 1:** Health educative text messages and appointment Short Message Service (SMS) reminders

*Health educative text messages*	
Hello! We would like to wish you a good day. The most common signs of cervical cancer are vaginal bleeding in between your periods or contact bleeding, for example after sexual intercourse. Another sign can be unusual discharge from the vagina. If you experience any health problems, it is important to visit your health clinic even before your next appointment. Thank you for reading our message.	
Hello! We would like to wish you a good day. Often women cannot feel cervical cancer and it shows no signs at an early stage. Therefore, it is important to go to screening even though you do not feel sick or have symptoms of cervical cancer. When cervical cancer is found at an early stage it can get treated. Thank you for reading our message.	
Hello! We are here to help you. Women who have Human Papillomavirus can still have sexual intercourse with their partner. Most often the body gets rid of the infection on its own. The most common signs of cervical cancer are vaginal bleeding in between your periods or contact bleeding, for example after sexual intercourse. Another sign can be unusual discharge from the vagina. When you go to screening, the nurse can check and see if your cervix looks healthy. Thank you for reading our message.	
*Appointment SMS reminders*	
Hello! It is time for your screening appointment. Go to your health clinic tomorrow. When you go to your screening appointment it will help you to stay healthy and free of cervical cancer. We are here to help you. Thank you for reading our message.	
Hello! You had a screening appointment 1 week ago. You are always welcome at the health clinic at any time and get screened for cervical cancer. When you go and get checked it will help you to stay healthy and free of cancer. It is important that you stay healthy for you and your family’s sake. Screening does not cost you anything. Thank you for receiving our messages. This is the last message. Thank you very much.	

**Fig. 2:**
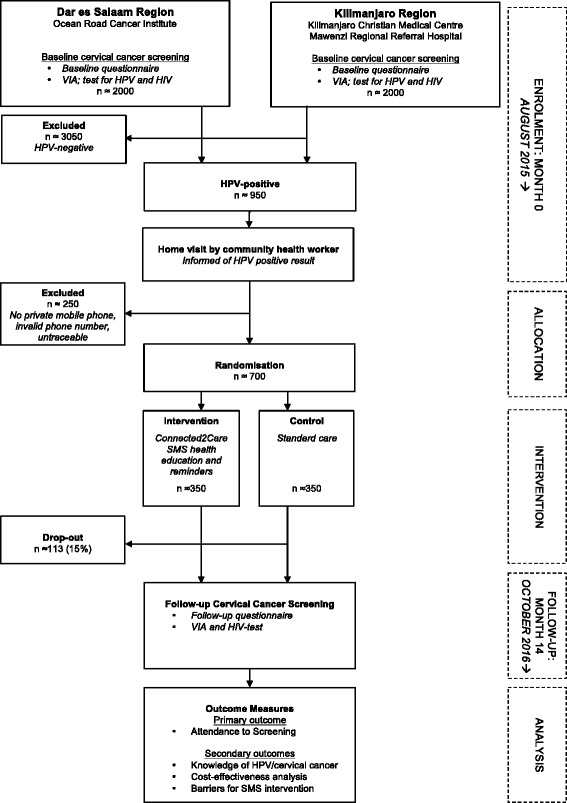
Trial Profile

An external IT consultant has developed a web-based SMS operating system for the study. The online operating system provides an overview of all text messages that have been, or are scheduled to be, sent and shows the following participant data: study number, mobile number, research site, enrolment and follow-up date, education, and age. The operating system has a delivery note feature that shows if there are any discrepancies between the number of messages sent to a participant and the number received. The text messages will be automatically dispatched through the operating system and will be one-directional, meaning that the recipient cannot reply to the SMS. The text messages will be sent with the ID ‘ElimuYaAfya’ (meaning ‘health education’ in Kiswahili). The SMSs required for the intervention are attained from the online Cloud SMS service ‘bulksms’ (www.bulksms.com). The SMS service supports all mobile operators in Tanzania. During the follow-up period, the SMS system will be monitored on a monthly basis by the first author and the external IT consultant in order to ensure that system is functioning and text messages are being dispatched as scheduled. The full trial profile is outlined in Fig. 2.

#### Pre-test of intervention

The messages were tested in a group of Tanzanian women prior to implementation and specific attention was given to the ethical aspects of receiving health information on mobile phones as well as the content and the timing of the messages. Two focus group discussions were held with six to eight women attending screening at ORCI and personal interviews were carried out with four women at KCMC. The pre-tests showed that apart from wanting to be informed about symptoms and the importance of screening, ‘everyday life’ information was important for the women (such as whether or not they could continue to have sexual intercourse). These aspects were incorporated into the text messages. The pre-tests further showed that the women preferred to receive the messages in the afternoon (prior to cooking dinner) or at night (after dinner time) as this was the ‘relaxation period of the day’. Since mobile networks in Tanzania can delay delivery of an SMS for up to 2 hours, it was decided that the messages should be sent at 5 p.m.

#### Intervention framework and procedures

At inclusion, all eligible and consenting women will be screened for cervical cancer using visual inspection with acetic acid (VIA), tested for HIV using a quick HIV-1/2 test (www.alere.com), and tested for HR HPV using the *care*HPV test® (www.qiagen.com). All women will be given a follow-up appointment date after 14 months where they will be re-screened using VIA and re-tested for HIV (if HIV negative at inclusion). Women who test VIA positive at inclusion will be treated on-site based on National Cervical Cancer Service Delivery Guidelines, which includes treatment with cryotherapy or loop electrosurgical excision procedure (LEEP). During examination, if manifest cancer is diagnosed, women will be managed based on the National Cervical Cancer Service Delivery Guidelines whereby a biopsy will be taken, and women will be referred for treatment at the oncology clinic at ORCI. The policy of the Tanzanian government is free cancer treatment for diagnosed patients whereby the government covers all costs. At inclusion, all consenting women will be interviewed by a trained nurse using a structured questionnaire. Questionnaire items will include sociodemographic and covariate information (including marital status, education level, HIV status, smoking and alcohol habits, sexual history), knowledge of screening and cervical cancer, as well as acceptance of receiving health educative mobile text messages. Contact information, including mobile phone number, will be registered on a separate contact information form.

### Study population and recruitment

The study is part of a larger research project, CONCEPT (Comprehensive Cervical Cancer Prevention in Tanzania) that started in August 2015 and finishes in December 2019 (Additional file 2).

CONCEPT is linked to the existing national cervical cancer screening programmes in Dar es Salaam and Kilimanjaro.

The study population consists of HPV-positive women who will be recruited as they visit the cervical cancer screening clinics, the reproductive health clinics or the CTCs at the study sites. To increase recruitment, fliers informing about the screening will be shared at churches and mosques close to the study sites.

#### Inclusion criteria

- Informed consent provided
- HPV-positive
- Age 25–60 years
- Owning a private mobile phone

#### Exclusion criteria

- Pregnant on the day of enrolment
- Menstruating on the day of enrolment
- Hysterectomy
- Diagnosed with cervical pre-cancer within past 12 months
- Diagnosed with cervical cancer
- Invalid mobile phone number
- Unreachable when trying to convey HPV-positive result

### Outcome measures

#### Primary outcome

The effect measure of the intervention is the 14-month follow-up attendance rate for HPV-positive women. Participants will be given a specific date for their follow-up appointment at enrolment. However, as women cannot attend screening during their menstrual period, women often show up for their appointment within ± 2 weeks of their appointment. Further extenuating factors, such as work or family obligations or transportation issues, are common in Tanzania. In order not to exclude any women who may be delayed for such reasons, attendance to follow-up will be measured as attending up to 30 days past the appointment date.

#### Secondary outcomes

The cost-effectiveness of the intervention is estimated through a conventional cost-effectiveness analysis based on the RCT [22]. Two incremental cost-effectiveness ratios (ICERs) will be calculated [23]; one with and one without the costs of HPV testing. One ICER will reflect all the costs related to this intervention (including HPV testing), and the other will reflect the basic costs of implementing an SMS intervention in a low-income setting (excluding HPV testing).

A 16-item true/false questionnaire regarding cervical cancer and screening will be used to measure the effect of the intervention on HPV-positive women’s level of knowledge. The questionnaire will be answered by all participants at baseline and by the intervention group at follow-up. The items reflect the health information that will be sent to the intervention group (Table 2).

**Table 2:** The 16-item questionnaire to measure knowledge of cervical cancer

Malaria (mosquito) causes cervical cancer	True ❏	False ❏
Pain during urination can be a sign of cervical cancer	True ❏	False ❏
Cervical cancer is the most common cancer disease among Tanzanian women	True ❏	False ❏
You can get cervical cancer from deep kissing	True ❏	False ❏
It is possible to prevent cervical cancer	True ❏	False ❏
Vaginal bleeding is the most common sign of cervical cancer	True ❏	False ❏
Too much sun can lead to cervical cancer	True ❏	False ❏
A cervical infection will always turn into cancer	True ❏	False ❏
HIV-positive women have higher risk of developing cervical cancer	True ❏	False ❏
Cervical cancer is often found at an early stage due to obvious symptoms	True ❏	False ❏
You can get cervical cancer from unprotected sexual intercourse	True ❏	False ❏
Screening can detect cervical infections so they do not develop into cancer	True ❏	False ❏
Cervical cancer is the main cause of cancer-related death among women	True ❏	False ❏
Cervical cancer is most common for women in their 20s	True ❏	False ❏
Itchiness in the vaginal area can be a sign of cervical cancer	True ❏	False ❏
A virus called ‘Human papilloma virus’ (HPV) causes cervical cancer	True ❏	False ❏

**Fig. 3:**
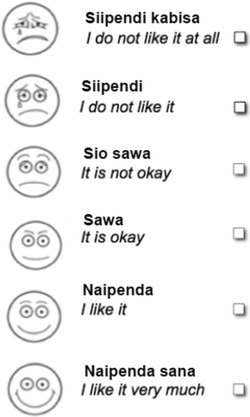
Likert scale to measure acceptability of text messages

Barriers for implementation of the intervention will be described in a mixed-methods sub-population study that combines quantitative and qualitative research methods [24, 25]. The quantitative component uses questionnaire items to measure acceptability, technical, and comprehension barriers. Acceptability will be measured through a six-point Likert scale using smiley faces at baseline and follow-up (Fig. 3). At follow-up, technical barriers and knowledge barriers will be measured through binary questionnaire items. The questionnaire outcomes will be supplemented by a qualitative sub-population study using individual interviews with women from the intervention group. There is no set outcome measure for the interviews as data are open-ended. The interviews will be conducted using a thematic semi-structured interview guide [26, 27].

### Analysis plan

#### Sample size

During the inclusion period, 4000 women will be screened for cervical cancer and it is expected that 950 (25%) women will test positive for HPV and that 25% (250) of these women will be excluded from the study due to there being no private mobile phone access, invalid mobile phone numbers, or they will be unreachable when trying to convey the HPV result. It is hypothesised that the intervention will increase attendance rates by 15%, and that 73% of the intervention group and 58% of the control will attend their follow-up appointment. A total dropout rate of 15% is expected. In order to detect an improvement in the intervention arm with a 95% probability and a power of 80%, it is estimated that 350 women will be needed in each study arm, i.e. a total sample size of 700. The sample size has been calculated based on the formulas and the nomogram in Altman’s paper [28].

#### Statistical methods

The primary analysis will be intention-to-treat and the intervention arm (SMS) will be compared to the control arm (standard care). Categorical variables will be expressed in frequency and percentage. A logistic regression analysis will be used to estimate the effect of the intervention; the 14-month follow-up attendance rate as the dependent binary variable and the intervention/controls as the independent variable. Potential confounders and effect modifiers (study site, region, educational level, age, HIV status, self-perceived health, previous cervical cancer screening attendance, marital status, cohabitors, religion) are addressed in a subsequent multiple logistic regression analysis. Results are expressed as odds ratios with 95% confidence intervals [29]. Demographic characteristics will be summarised using descriptive statistics. Continuous variables will be expressed as number of observed values, mean ± standard deviation, median (range). Trial results will be reported by use of the Consolidated Standards of reporting Trial (CONSORT) criteria (www.equator-network.org).

### Additional analysis

#### Cost-effectiveness evaluation

The cost-effectiveness analysis will include a 14-month time perspective corresponding to the duration from inclusion until the follow-up contact. The effect measure will be the same as in the RCT; 14-month follow-up attendance rates for HPV-positive women. The costs of the intervention will be estimated in US$ and include the HPV test, resource-use related to the technical development of the intervention, costs/SMS, and salaries to health personnel. Data on resource-use will be collected for each participating site based on available budget and financial information. Further, the indirect cost for not attending follow-up, i.e. the increased risk of developing cervical cancer, will be addressed.

#### Knowledge of screening and cervical cancer

A comparative analysis will be made between the number and percentage of correct answers on the 16-item true/false questionnaire at baseline and follow-up.

#### Mixed-methods sub-study

To obtain a more comprehensive account of barriers and enhance the overall application of the intervention, a qualitative component will supplement the questionnaire items used in the RCT [24]. After the intervention period, approximately 15 semi-structured, individual interviews will be conducted with participants from the intervention group. The qualitative data will be analysed according to a condensation of meaning analysis where natural units of meaning are detected and coded into central themes [24, 26, 27, 30]. The quantitative data analysis of the statistical findings from the RCT will entail a descriptive, comparative analysis. The quantitative and qualitative data will not be fully integrated during the interpretation of results. However, study results will be compared and conclusions will reflect what has been learned in a combination of the two studies [24].

### Data management

To ensure anonymity, all study participants will be given a unique study ID at inclusion and at follow-up. Informed consent and patient contact information will be stored in secured, separate folders at ORCI and KCMC. Copies of the contact information of HPV-positive women will be shared with research assistants so they are able to trace and visit the women. To monitor the progress of enrolment, study ID, phone number, HPV, HIV, and VIA status will be entered into an online electronic Excel® sheet on a weekly basis. To monitor the progress of follow-up, baseline study ID, follow-up-study ID, a date for a scheduled appointment and a date for actual appointment attendance will be entered into an online electronic Excel® sheet on a weekly basis. If the actual attendance date is within 30 days of the scheduled appointment date, the participant will be registered as a ‘turn-up’, otherwise as a ‘no-show’.

Data for the SMS intervention will be extracted from the Excel® sheet and uploaded to the online operating system manually once a month. The system has a private domain and a one-direction encryption password is required to access the system. The system runs on a Cloud service provided by Linode (www.linode.com). The domain name http*://*connected2care.org is registered from Linode, and the system is accessed through this domain. All data will be backed up on a weekly basis.

Data will be entered and managed through the Research Electronic Data Capture (REDCap) (www.project-redcap.org) and then exported to STATA v15 (www.stata.com). REDCap ensures that data are stored at a secured, web-based server at the University of Southern Denmark, Odense. Only the first author and a data manager from the University of Southern Denmark will have access to the data. Electronic audio files will be stored on an online secured server at the University of Southern Denmark. Transcriptions will be anonymised.

### Challenges in study implementation

All text messages will be sent using an online Cloud SMS service. Originally, a stationary server with a pre-paid SMS service located at ORCI was used for the intervention. However, 6 months into the intervention period random checks showed that the server was unstable and not dispatching the text messages as according to the study plan. Therefore, the study team transitioned to the online Cloud SMS service and re-started the intervention 6 months into the study. Already-enrolled patients kept their randomisation status and may have received up to four text messages twice. We will examine the effect on the transition on this sub-group during the analysis.

Initially, only two study sites were part of the study, ORCI and KCMC. However, due to delays in inclusion, it was decided to add another site to the study in 2016, Mawenzi Regional Referral Hospital. Further, the period from the initial screening until women have been informed of their positive HPV result has at times also been prolonged, which has entailed that the first number of text messages tend to bulk (are received at one time instead of once a month). We will examine the effect of this issue during a sub-group analysis.

## Discussion

This study protocol describes an RCT that is designed to improve attendance to cervical cancer screening follow-up appointments in an African setting through educational mobile text messages and appointment SMS reminders. Additional to the RCT, the study investigates the cost-effectiveness of the intervention through a conventional cost-effectiveness analysis as well as barriers for implementation through a mixed-methods sub-population study. The study findings will provide valuable information of the real-world feasibility of implementing an SMS intervention in a low-income setting, which can be a valuable tool for increasing the effectiveness of screening services. There are several elements of special importance in the study that are worth outlining.

The study has had several implementation issues, i.e. challenges in relation to setting up a stable SMS service and delays during inclusion. Despite LICs, such as Tanzania, having moved into the mobile era, networks and Internet tend to be unstable, which is challenging for a mobile intervention such as *Connected2Care*. These issues must be encountered for in future planning of an SMS intervention study or large-scale implementation in a similar setting.

The study tests women for HPV in addition to the regular VIA examination, which is standard care in Tanzania. This test is introduced because research has shown that the sensitivity of VIA is low compared to HPV testing [6]. It is important that the study participants understand the meaning of the test and the health education that they receive on their mobile phones. At inclusion, all women are, therefore, given health education about HPV and how it relates to cervical cancer as well as information about the mobile intervention. A protocol has been developed for how community health workers deliver HPV-positive results to lay persons in an ethically appropriate and consistent manner, and how they will address any concerns the women may have in relation to their result. Despite these precautionary measures, there is a risk that study participants misinterpret an HPV-positive result as having cervical cancer or do not understand the mHealth education due to low health literacy [31, 32]. This could have social and mental health consequences as research shows that cervical cancer can be perceived as a death sentence [32, 33]. Thirty days past the follow-up appointment date, hospital nurses trace women who have not attended their appointment. The women are encouraged to come to the clinic for screening and the importance of the follow-up appointment is explained. For ethical concerns, women who do not have access to a private mobile phone are excluded from this study, as text messages are considered too sensitive for shared mobile phones. A possible consequence of this is selection bias, since poorer women may not have access to a private mobile phone and, therefore, may be excluded.

Despite these issues, if, as expected, the mobile intervention increases attendance to follow-up appointments among women who have tested positive for HR HPV, more women will be ensured an earlier follow-up, which increases the chance of successful treatment and survival. Hereby, this study may help to improve the prevention of cervical cancer in Tanzania.

### Study duration/trial status

It is anticipated that the total duration of the study will be 3.5 years. The inclusion period started in August 2015 and is anticipated to finish in the second half of 2017. The follow-up period is 14 months. End of study will be in 2019. The study has been registered at ClinicalTrials.gov: NCT02509702.

## Additional files

Additional file 1: SPIRIT Checklist. Complete SPIRIT Checklist. (PDF 887 kb)

Additional file 2: CONCEPT protocol. Protocol for the overall CONCEPT protocol. Trial is listed under Work Package 4. (PDF 424 kb)

## Abbreviations

CONCEPT: Comprehensive Cervical Cancer Prevention in Tanzania
CTC: Care and treatment clinics
HIV: Human immunodeficiency virus
HPV: Human Papillomavirus
HR: High-risk
ICER: Incremental cost-effectiveness ratio
KCMC: Kilimanjaro Christian Medical Centre
LEEP: Loop electrosurgical excision procedure
LICs: Low-income countries
mHealth: Mobile Health
ORCI: Ocean Road Cancer Institute
RCT: Randomised controlled trial
REDCap: Research Electronic Data Capture
SMS: Short Message Service
VIA: Visual inspection with acid
