# Supplementary material for: Text messages to increase attendance to follow-up cervical cancer screening appointments among HPV-positive Tanzanian women (Connected2Care): study protocol for a randomised controlled trial
Source: Trials. 2017 Nov 21;18:555. doi: 10.1186/s13063-017-2215-x (PMC5698928; doi:10.1186/s13063-017-2215-x)
Supplement: Supplementary file 1 — SPIRIT Checklist. Complete SPIRIT Checklist. (PDF 887 kb) [file 13063_2017_2215_MOESM1_ESM.pdf]

STANDARD PROTOCOL ITEMS: RECOMMENDATIONS FOR INTERVENTIONAL TRIALS

SPIRIT 2013 Checklist:

Section/item	Item No	Description
**Administrative information**		
**Title**	**1**	**Descriptive title identifying the study design, population, interventions, and, if applicable, trial acronym**<br><br>Text Messages to Increase Attendance to Follow-up Cervical Cancer Screening Appointments among HPV Positive Tanzanian Women. The *Connected2Care* Randomised Controlled Trial Study Protocol.
**Trial registration**	**2a**	**Trial identifier and registry name. If not yet registered, name of intended registry**<br><br>ClinicalTrials.gov: NCT02509702

2b All items from the World Health Organization Trial Registration Data Set

DATA CATEGORY	INFORMATION
Primary registry and trial identifying number	ClinicalTrials.gov<br>NCT02509702
Date of registration in primary registry	15 June 2015
Secondary identifying numbers	-
Source(s) of monetary or material support	The Danish International Development Agency<br>University of Southern Denmark
Primary sponsor	The Danish International Development Agency
Secondary sponsor(s)	University of Southern Denmark
Contact for public queries	DSL, MPH, PhD student [+4561666564] [dsondergaard@health.sdu.dk]
Contact for scientific queries	DSL, MPH, PhD student<br>Department of Obstetrics and Gynaecology, Odense University Hospital, Denmark
Public title	*Connect2Care:* How to Increase Attendance to Follow-up Cervical Cancer Screening in Tanzania through Text Messages
Scientific title	Text Messages to Increase Attendance to Follow-up Cervical Cancer Screening Appointments among HPV Positive Tanzanian Women. The *Connected2Care* Randomised Controlled Trial Study Protocol.
Countries of recruitment	Tanzania
Health condition(s) or problem(s) studied	Cervical Cancer
Intervention(s)	*Active comparator:* SMS intervention (ten health educative text messages and five SMS-reminders).<br>*Passive comparator:* Standard care (follow-up appointment 14 months after the initial screening appointment)
Key inclusion and exclusion criteria	Ages eligible for study: 25 – 60 years<br>Sexes eligible for study: Female<br>Inclusion criteria: Informed consent, HPV positive, private mobile phone.<br>Exclusion criteria: Pregnant on day of enrolment, menstruating on day of enrolment, hysterectomy, diagnosed with cervical pre-cancer within past 12 months, diagnosed with cervical cancer, invalid mobile phone number, unreachable when trying to convey HPV positive result.
Study type	Interventional<br>Allocation: randomised<br>Intervention model: parallel assignment<br>Masking: none<br>Primary purpose: prevention
Date of first enrolment	August 2015
Target sample size	700
Recruitment status	Recruiting
Primary outcome(s)	14-month follow-up attendance rate for HPV positive women
Key secondary outcomes	*Cost-effectiveness:* measured through Incremental Cost-Effectiveness Ratios<br>*Knowledge of screening and cervical cancer:* measured through a 16-item true/false questionnaire<br>*Barriers for mHealth implementation:* measured in mixed method sub-study through a 6-point Likert Scale and semi-structured interviews.

**Protocol version**	**3**	**Date and version identifier**<br>*Issue Date:* March 2016<br>*Protocol Amendment Number:* 02<br>*Authors:* DSL, MSA, JDM, RM, SKK, VR

Revision Chronology:

June 2015	Original
October 2015	Amendment no. 01: Elaboration of ethical considerations<br>Changes in section 3.0 'study population' regarding the exclusion criterion of access to mobile phone (from shared family phone to private)<br>Changes in Section 6.0 'ethical considerations' regarding possible psychological consequences of receiving mobile health messages and how to convey HPV positive results to participants.<br>Additional changes: elaboration of link between HPV and cervical cancer
March 2016	Amendment 02: Additional study sites<br>Changes in section 3 'study population' regarding adding additional study sites and further elaboration of exclusion criteria.<br>Changes in section 4.3 'Acceptability of Connected to Care' regarding elaboration of mixed method study design, study sites and focus area.

**Funding**	**4**	**Sources and types of financial, material, and other support**<br>This trial is part of the research project Comprehensive Cervical Cancer Prevention in Tanzania (CONCEPT), which is a five-year research project. The Danish International Development Agency (Danida) is funding the run-in costs for the CONCEPT project, which goes on from January 2015 to December 2019. The funding from Danida covers all costs for developing and implementing this RCT, which goes on from August 2015 to July 2018. This includes costs for technical development of the mobile intervention, transport and equipment expenses as well as 2/3 of the payment for the PhD student responsible for conducting the trail. The University of Southern Denmark finances the remaining 1/3 of the payment of the PhD student through a Faculty Scholarship.

Roles and responsibilities

5a **Names, affiliations, and roles of protocol contributors**

Ditte S Linde[a, b, c, *], Marianne S Andersen[d], Julius D Mwaiselage[e], Rachel Manongi[f], Susanne K Kjaer[g, h], Vibeke Rasch[a,b]

[a] Department of Obstetrics and Gynaecology, Odense University Hospital, Odense, Denmark
[b] Institute of Clinical Research, University of Southern Denmark, Odense, Denmark. Email: dsondergaard@health.sdu.dk and vrasch@health.sdu.dk
[c] OPEN, Odense Patient Data Explorative Network, Odense University Hospital, Odense, Denmark
[d] Department of Medical Endocrinology, Odense University Hospital, Odense, Denmark. Email: Marianne.Andersen1@rsyd.dk
[e] Department for Cancer Prevention Services, Ocean Road Cancer Institute, Dar es Salaam, Tanzania. Email: Julius.mwaiselage@orci.or.tz
[f] Institute of Public Health, Kilimanjaro Christian Medical University College, Moshi, Tanzania. Email: naeraheli@gmail.com
[g] Department of Gynaecology, Rigshospitalet University Hospital, Copenhagen, Denmark. Email: susanne@cancer.dk
[h] Department of Virus, Lifestyle and Genes, Danish Cancer Society Research Center, Copenhagen, Denmark

Authors' contributions:
DSL drafted the manuscript and contributed to conceptualising and designing the study. VR and SKK contributed to conceptualising and designing the study and critically revised the manuscript. MSA contributed to the study methods/design and critically revised the manuscript. JDM and RM contributed to conceptualising and designing the study. All authors contributed to refinement of the study protocol and approved the final manuscript.

5b **Name and contact information for the trial sponsor**

Trial Sponsor: Ocean Road Cancer Institute
Sponsor's Reference: Executive Director
Contact name: Julius Mwaiselage
Address: Barack Obama Drive, P.O. Box 3592, Dar es Salaam, Tanzania
Telephone: +255 784 764 412/ +255 754 764 412
Email: Julius.mwaiselage@orci.or.tz

5c **Role of study sponsor and funders, if any, in study design; collection, management, analysis, and interpretation of data; writing of the report; and the decision to submit the report for publication, including whether they will have ultimate authority over any of these activities**

The funding sources have had no role in the design of the study and will not have any role during its execution, analysis, interpretation of the data, or decision to submit results.

5d Composition, roles, and responsibilities of the coordinating centre, steering committee, endpoint adjudication committee, data management team, and other individuals or groups overseeing the trial, if applicable (see Item 21a for data monitoring committee)

Organisational structure and responsibilities

Principal Investigator (JDM in cooperation with DSL)
Design and conduct of study
Preparation of protocol and revisions
Study planning
Randomisation

Steering Committee (VR, SKK, JDM, RH)
Design and study planning
Overall design of CONCEPT study
Agreement of final protocol
Budget administration
Organisation of steering committee meetings
Advice for lead investigators
Ongoing review of study process

Lead Investigator (DSL)

Introduction

**Background and rationale**	**6a**	**Description of research question and justification for undertaking the trial, including summary of relevant studies (published and unpublished) examining benefits and harms for each intervention**

Background

Human Papilloma Virus (HPV) is the most common sexually transmitted infection in the world and approximately 80% of all sexually active persons will be infected with HPV at some point during their lifetime[1,2]. Persistent High-Risk (HR) HPV infection is a necessary first step in developing cervical precancerous lesions. A minority of these lesions progress to invasive cervical cancer over 10-20 years. The natural history of infection to cancer is complex, and many factors influence this, including the type of HPV, the number of sexual partners, parity, other sexually transmitted agents, and host susceptibility[3]. Cervical cancer often affects women of reproductive age and is a major health challenge in low-income countries (LICs) such as Tanzania where it accounts for 38% (n=7300) of all female cancers, and 34% (n=4200) of all female cancer-related deaths[1,4]. HIV-infected women are at high risk of contracting HPV infection and developing cervical cancer. Further, cervical cancer screening is limited in Tanzania and the screening uptake is poor due to lack of knowledge of the disease and its prevention. These factors are contributing to the large cervical cancer burden in Tanzania, and the disease is a public health concern with enormous social and economic impact[1–3,5–10]. It is yet to be estimated to which degree women, who have attended screening and have had abnormal test results, will attend follow-up screening appointments in East-African populations.

As many other African nations, Tanzania is skyrocketing into the mobile era. By the end of 2014, 75% of the population (34 million) had a mobile subscription[5]. The opportunity to give mobile technology a formal role in health care is increasingly recognised[11]. The Ministry of Health in Tanzania has Mobile Health (mHealth) as part of its Electronic Health (eHealth) strategy from 2013 – 2018[12]. Randomised Control Trials (RCTs) have documented that mobile technology can have a positive effect on both patients' and health personnel's behaviours in high as well as in low income settings[11,13–20]. In East Africa, mHealth interventions have proven to increase attendance with skilled delivery staff among pregnant women (by 13%); adherence to anti-retroviral therapy among HIV-positive patients (by 12-13%); attendance to post-operative clinic visits among men who have been circumcised for HIV prevention (by 5.7%); and correct malaria-case management among health personnel (by 23-24%)[13–16,20]. However, mHealth initiatives are still in their early days in LIC, and for full effect it is key to consider literacy, cultural, technical and scalability issues during implementation[11].

To our knowledge, no RCTs have previously tested the degree to which text messages can improve cervical cancer screening behaviour and follow-up of women who have had abnormal screening results. However, RCTs from high-income countries have shown that educative text messages and SMS reminders can significantly improve other types of cancer preventive behaviour, for example self-reported skin cancer prevention; breast self-examinations and breast cancer screening attendance [17–19].

6b **Explanation for choice of comparators**

Active comparator: SMS intervention (10 health educative text messages and five SMS-reminders sent over a period of 10 months).

Passive comparator: Standard of care (follow-up appointment 14 months after the initial screening appointment given on a physical appointment card to the woman)

Reason for choice of comparators: The intervention is compared to standard care as this the current recommended practice in Tanzania.

Objectives **7** **Specific objectives or hypotheses**

Hypothesis:

The SMS intervention will increase attendance to follow-up check-up screening appointments among HPV positive women by 15%.

Primary objective:

- To assess the effect of an SMS intervention on HPV positive women's attendance for cervical cancer follow-up appointments at 14 months compared to standard care.

Secondary objectives:

- To estimate the cost-effectiveness of an SMS intervention
- To assess the effect of an SMS intervention on HPV positive women's knowledge of cervical cancer and screening
- To understand barriers against the implementation of an SMS intervention in Tanzania in a mixed methods sub-population study.

Trial design **8** **Description of trial design including type of trial (eg, parallel group, crossover, factorial, single group), allocation ratio, and framework (eg, superiority, equivalence, noninferiority, exploratory)**

Connected2Care is a randomised, three-site, control trial with two parallel groups and a primary endpoint of attendance to cervical cancer screening follow-up appointment among HPV positive women at 14 months. HPV positive women are randomly assigned 1:1 to a mobile phone intervention or a control group. The control group receives standard of care, which is a check-up appointment 14 months after the initial screening appointment given on a physical appointment card to the woman. On top of standard of care, the intervention group receives the SMS intervention.

The framework of the trial is to test superiority of sending HPV positive women health informative text messages and SMS-reminders over standard of care.

Methods: Participants, interventions, and outcomes

Study setting **9** **Description of study settings (eg, community clinic, academic hospital) and list of countries where data will be collected. Reference to where list of study sites can be obtained**

The study is conducted at three health facilities in Tanzania – one in the Dar es Salaam Region and two in the Kilimanjaro Region. All sites are urban or semi-urban areas. The study site in Dar es Salaam is the cervical cancer screening clinic at Ocean Road Cancer Institute (ORCI). The two study sites in the Kilimanjaro Region are the reproductive health clinics and the Care and Treatment Clinics (CTCs) at (1) Kilimanjaro Christian Medical Centre (KCMC), and (2) Mawenzi Regional Referral Hospital in Moshi.

List of sites
Ocean Road Cancer Institute
Barack Obama Drive
P.O. Box 3592
Dar es Salaam, Tanzania

Kilimanjaro Christian Medical Centre
P.O. Box 3010
Moshi, Tanzania

Mawenzi Regional Referral Hospital
P.O. Box 3010
Moshi, Tanzania

Eligibility criteria **10** **Inclusion and exclusion criteria for participants. If applicable, eligibility criteria for study centres and individuals who will perform the interventions (eg, surgeons, psychotherapists)**

Inclusion criteria

- Informed consent
- HPV positive
- Age 25 – 60 years
- Private mobile phone

Exclusion criteria

- Pregnant on day of enrolment
- Menstruating on day of enrolment
- Hysterectomy
- Diagnosed with cervical pre-cancer within past 12 months
- Diagnosed with cervical cancer
- Invalid mobile phone number
- Unreachable when trying to convey HPV positive result

**Interventions**	**11a**	**Interventions for each group with sufficient detail to allow replication, including how and when they will be administered**<br><br>The intervention group receives a total of 15 text messages over a period of 10 months. There are two types of text messages: (1) educational text messages; and (2) SMS reminders for the follow-up appointment. There are 10 educational messages, which are sent once a month that includes risk factors, common symptoms, and screening information. The SMS reminders inform the participant of their appointment date and encourage participants who have missed their follow-up appointment to attend the screening. The SMS-reminders are sent two weeks, one week, and one day prior to the appointment date as well as one day and one week post the appointment date.
	**11b**	**Criteria for discontinuing or modifying allocated interventions for a given trial participant (eg, drug dose change in response to harms, participant request, or improving/worsening disease)**<br><br>Technical Disturbances<br><br>All text messages are sent using an online cloud SMS service. Initially, a stationary server with a pre-paid SMS service was used for the intervention. However, six months into the intervention period random checks showed that the server was unstable and not dispatching the text messages as according to the study plan. Therefore, the study team transitioned to the online cloud SMS service and re-started the intervention six months into the study. Already enrolled patients kept their randomisation status and may have received up four text messages twice. We will examine the effect on the transition on this sub-group during the analysis.
	**11c**	**Strategies to improve adherence to intervention protocols, and any procedures for monitoring adherence (eg, drug tablet return, laboratory tests)**<br><br>The online cloud SMS service has a delivery note feature that shows if there are any discrepancies between the number of messages sent to a participant and the number of messages received. Hereby, it is possible to estimate whether or not the intervention (the number of SMS' received) differs among study participants.
	**11d**	**Relevant concomitant care and interventions that are permitted or prohibited during the trial**<br><br>Not Relevant

**Outcomes**	**12**	**Primary, secondary, and other outcomes, including the specific measurement variable (eg, systolic blood pressure), analysis metric (eg, change from baseline, final value, time to event), method of aggregation (eg, median, proportion), and time point for each outcome. Explanation of the clinical relevance of chosen efficacy and harm outcomes is strongly recommended**

Primary outcome measure

The effect measure of the intervention is the 14-month follow-up attendance rate for HPV positive women. Participants are given a specific date for their follow-up appointment at enrolment. However, as women cannot attend screening during their menstrual period, women often show up for their appointment -/+ 2 weeks of their appointment. In order not to exclude any women who may be delayed for such reasons, attendance to follow-up is measured as turn-up up to 30 days past the appointment date.

Secondary outcome measure

Cost-effectiveness

The cost-effectiveness of the intervention is estimated through a conventional Cost-Effectiveness Analysis (CEA) based on the RCT. Two Incremental Cost-Effectiveness Ratios (ICERs) are calculated; one with and one without the costs of HPV testing. Hereby, one ICER reflects all the costs related to this intervention (including HPV testing), and one reflects the basic costs of implementing a mHealth initiative in a low-income setting (excluding HPV testing).

Knowledge of HPV, cervical cancer, and screening

A 16-item true/false questionnaire regarding cervical cancer and screening is used to measure the effect of the intervention on HPV positive women's level of knowledge. The questionnaire is answered by all participants at baseline and by the intervention group at follow-up. The items reflect the health information that is sent to the intervention group.

Barriers for implementation of mHealth intervention

Barriers for implementation of the intervention is described in a mixed methods sub-study that combines quantitative and qualitative research methods. The quantitative component uses questionnaire items to measure acceptability, technical, and comprehension barriers. Acceptability is measured through a 6-point Likert-scale using smiley faces at baseline and follow-up. At follow-up, technical barriers and knowledge barriers are measured through binary questionnaire items.

The questionnaire outcomes are supplemented by a qualitative sub-study that use individual interviews with HPV positive women. There is no set outcome measure for the interviews as data is open-ended. The interviews are conducted using a thematic semi-structured interview guide.

**Participant timeline**	**13**	**Time schedule of enrolment, interventions (including any run-ins and washouts), assessments, and visits for participants. A schematic diagram is highly recommended (see Figure)**

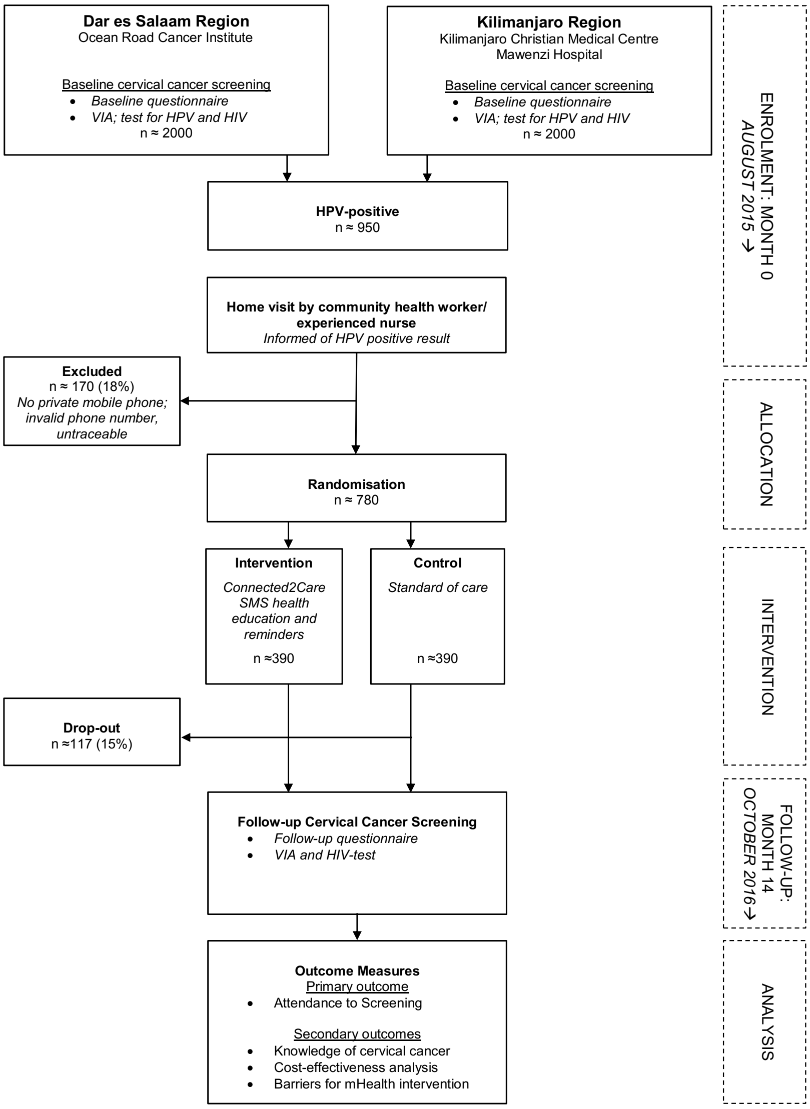

**Sample size**	**14**	**Estimated number of participants needed to achieve study objectives and how it was determined, including clinical and statistical assumptions supporting any sample size calculations**

It is hypothesised that the intervention will increase attendance rates by 15%. In order to detect an improvement in the intervention arm with a 95% probability and a power of 80%, it is estimated that 350 women are needed in each study arm. According to the CONCEPT study protocol, approximately 4000 women will be screened for cervical cancer in the inclusion period, and it is expected that 24% of these test positive for HPV, which is equivalent to 950 women. The trial experiences for the first 12 months show that approximately 25% of the women test positive for HPV, 14% of these are excluded due to no mobile phone access or incorrect phone numbers. Given that 4000 women will be screened for cervical within the inclusion period, it is anticipated that the pool of eligible study participants will be sufficient to reach the target of a sample size of 700.

**Recruitment**	**15**	**Strategies for achieving adequate participant enrolment to reach target sample size**

The study is part of a larger research project, CONCEPT (Comprehensive Cervical Cancer Prevention in Tanzania) that started in August 2015 and finishes in December 2019.
CONCEPT is linked to the existing national cervical cancer screening programmes in Dar es Salaam and Kilimanjaro.

The study population consists of Tanzanian women that are HPV positive, aged 25-60 years living in the proximity of Dar es Salaam and Kilimanjaro. Study participants are recruited as they visit the cervical cancer screening clinics, the reproductive health clinics, or the CTCs at the study sites. To increase recruitment, fliers informing about the screening are shared at churches and mosques close to the study sites.

Methods: Assignment of interventions (for controlled trials)

Allocation:

**Sequence generation**	**16a**	**Method of generating the allocation sequence (eg, computer-generated random numbers), and list of any factors for stratification. To reduce predictability of a random sequence, details of any planned restriction (eg, blocking) should be provided in a separate document that is unavailable to those who enrol participants or assign interventions**<br><br>Participants will be randomly assigned to either the intervention group or the control group with a 1:1 allocation. The randomisation is non-blinded and occurs automatically when participant data are uploaded to the SMS system through an incorporated algorithm in the system.
**Allocation concealment mechanism**	**16b**	**Mechanism of implementing the allocation sequence (eg, central telephone; sequentially numbered, opaque, sealed envelopes), describing any steps to conceal the sequence until interventions are assigned**<br><br>The SMS system has been developed by an external IT-consultant. Only the IT-consultant knows the algorithm that allocates participants into the intervention or control group
**Implementatio n**	**16c**	**Who will generate the allocation sequence, who will enrol participants, and who will assign participants to interventions**<br><br>All women who give consent for participation and fulfil the inclusion criteria will be randomised. Participants are enrolled by cancer screening nurses and informed about their positive HPV status through a home visit by a community nurse. Once the community nurse confirms to the primary investigator that the HPV positive woman has been informed of her test result, the primary investigator uploads participant data to the SMS system and then the randomisation occurs. Participant data that are uploaded to the SMS system is: Study number; mobile number; research site; enrolment and follow-up date; education; and age. The SMS system shows all uploaded data and the randomisation status of each participants; “1” for intervention group and “0” for control group.
**Blinding (masking)**	**17a**	**Who will be blinded after assignment to interventions (eg, trial participants, care providers, outcome assessors, data analysts), and how**<br><br>The study is non-blinded

	**17b**	**If blinded, circumstances under which unblinding is permissible, and procedure for revealing a participant's allocated intervention during the trial**
		NA

Methods: Data collection, management, and analysis

**Data collection methods**	**18a**	**Plans for assessment and collection of outcome, baseline, and other trial data, including any related processes to promote data quality (eg, duplicate measurements, training of assessors) and a description of study instruments (eg, questionnaires, laboratory tests) along with their reliability and validity, if known. Reference to where data collection forms can be found, if not in the protocol**

At inclusion, all women that are eligible and consenting to participate in the overall CONCEPT project are screened for cervical cancer using Visual Inspection with Acid (VIA), tested for HIV using a quick HIV 1/2-test, and tested for High-Risk (HR) HPV using the Care HPV technique. All women are screened by trained nurses and a follow-up appointment date after 14 months where they will be re-screened using VIA and re-tested for HIV (if HIV negative at inclusion). Women who were VIA positive at inclusion are treated onsite based on National Cervical Cancer Service Delivery Guidelines, which includes treatment with cryo therapy or Loop Electrosurgical Excision Procedure (LEEP). During examination, if manifest cancer is diagnosed, women are also managed based on the National Cervical Cancer Service Delivery Guidelines whereby a biopsy is taken, and women are referred for treatment at the oncology clinic at ORCI. The policy of the Tanzanian government is free cancer treatment for diagnosed patients whereby the government covers all costs. At the end of each day HPV samples are transported to a local laboratory where they tested by laboratory technicians within 30 days following the Care HPV procedure and training.

At inclusion, all consenting women are interviewed by a trained nurse using a structured validated questionnaire. Questionnaire items includes socio-demographic and covariate information (marital status; education level; HIV-status; smoking- and alcohol habits; sexual history; etc.), knowledge of screening and cervical cancer as well as acceptance of receiving health educative mobile messages. Contact information including mobile phone number is registered on a separate contact information form.

18b Plans to promote participant retention and complete follow-up, including list of any outcome data to be collected for participants who discontinue or deviate from intervention protocols

Out of ethical concerns, all eligible consenting women have to be informed about their positive HPV result before being enrolled into the study. Once the HPV results are ready, a community nurse is therefore given a copy of the contact information form and visits the woman at home to inform her of her result as well as the possibility of receiving SMS' until her follow-up appointment. The nurse encourages the woman to attend her follow-up appointment at 14 months. Once 30 days have passed the follow-up appointment date, the woman is by definition lost to follow-up. However, out of ethical concerns as well as other study objectives in the overall CONCEPT study the woman is being traced for follow-up once past the 30 day mark. Firstly, a nurse tries to call the woman three times and encourage her to come to the follow-up appointment as well as inform her that her travelling costs will be disbursed if she comes. If the woman does not show up within one week, secondly a nurse will visit the woman at home and encourage her to come to the clinic. If the woman does not turn up for her follow-up appointment within another week, a nurse visits the woman at home and conducts the follow-up appointment at the woman's house if she consents to it.

**Data management**	**19**	**Plans for data entry, coding, security, and storage, including any related processes to promote data quality (eg, double data entry; range checks for data values). Reference to where details of data management procedures can be found, if not in the protocol**

To ensure anonymity, all study participants are given a unique study-id at inclusion and at follow-up. Informed consent and patient contact information are stored in secured, separate folders at ORCI and KCMC. Copies of the contact information of HPV positive women are shared with research assistants so they are able to trace and visit the women. To monitor the progress of the data collection, the study-id, phone number, HPV-, HIV-, and VIA status are entered into an online electronic excel-sheet on a weekly basis.

Data for the SMS intervention are extracted from the excel-sheet and uploaded to the online operating system manually once a month. The system has a private domain and a one direction encryption password is required to access the system. The system runs on a cloud service provided by Linode (Cloud Server). The domain name *http://connected2care.org* has been registered from Linode until March 2018, and the system is accessed through this domain. All data are backed up on weekly basis.

Data are entered and managed through the Research Electronic Data Capture (REDCap) and then exported to STATA v14. REDCap ensures that data are stored at a secured, web-based server at the University of Southern Denmark. Only the primary investigator and data manager from the University of Southern Denmark have access to the data.

Electronic audio files are stored on an online secured.

**Statistical methods**	**20a**	**Statistical methods for analysing primary and secondary outcomes. Reference to where other details of the statistical analysis plan can be found, if not in the protocol**

Primary analysis

In the primary analysis the intervention arm (SMS) will be compared to the control arm (standard of care). Binary and categorical variables are expressed in frequency and percentage. A logistic regression analysis is used to estimate the effect of the intervention; the 14-month follow-up attendance rate as the dependent binary variable and the intervention/controls as the independent variable. Potential confounders (region, educational level, age, HIV-status, etc.) are addressed in a subsequent multiple logistic regression analysis. Results are expressed as Odds Ratios with 95% confidence intervals. Demographic characteristics are summarised using descriptive statistics. Continuous variables are expressed as number of observed values, mean ± SD, median (range).

Additional analysis

Cost-effectiveness evaluation

The cost-effectiveness analysis is conducted with the perspective of the Tanzanian health care system and includes a 14-months' time perspective corresponding to the duration from inclusion until the follow-up contact. The effect measure is the same as in the RCT; 14-months follow-up attendance rates for HPV positive women. The costs of the intervention are estimated in US$ and include the HPV test, resource-use related to the technical development of the intervention, costs/ SMS, and salaries to health personnel. Data on resource-use are collected for each participating site based on available budget and financial information.

Knowledge of screening and cervical cancer

A comparative analysis is made between the number and percentage of correct answers on the 16-item true/false questionnaire at baseline and follow-up.

Mixed Methods sub-study

To get a more comprehensive account of barriers and enhance the overall application of the intervention, a qualitative component supplements the questionnaire items used in the RCT. Post intervention period, approximately 20 semi-structured, individual interviews are conducted with participants from the intervention group. The qualitative data are analysed according to a condensation of meaning analysis where natural units of meanings are detected and coded into central themes. The quantitative data analysis of the statistical findings from the RCT entails a descriptive, comparative analysis. The quantitative and qualitative data will not be fully integrated during the interpretation of results. However, study results will be compared and conclusions will reflect what has been learned in a combination of the two studies.

	20b	**Methods for any additional analyses (eg, subgroup and adjusted analyses)**<br><br>See 20a
	20c	**Definition of analysis population relating to protocol non-adherence (eg, as randomised analysis), and any statistical methods to handle missing data (eg, multiple imputation)**<br><br>Analyse descriptive variables of show-ups versus no-shows for follow-up appointment. Analyses on site levels.
**Methods: Monitoring**		
**Data monitoring**	21a	**Composition of data monitoring committee (DMC); summary of its role and reporting structure; statement of whether it is independent from the sponsor and competing interests; and reference to where further details about its charter can be found, if not in the protocol. Alternatively, an explanation of why a DMC is not needed**<br><br>There is a CONCEPT steering Committee that oversees the overall CONCEPT study. See 5d.
	21b	**Description of any interim analyses and stopping guidelines, including who will have access to these interim results and make the final decision to terminate the trial**<br><br>Not relevant
**Harms**	22	**Plans for collecting, assessing, reporting, and managing solicited and spontaneously reported adverse events and other unintended effects of trial interventions or trial conduct**<br>• Primary analysis of attendance to follow-up among intervention arm and control arm<br>• Acceptability of SMS' through questionnaire items at follow-up
**Auditing**	23	**Frequency and procedures for auditing trial conduct, if any, and whether the process will be independent from investigators and the sponsor**<br><br>• Pre-test of SMS-system prior to implementation<br>• SMS delivery feature incorporated into SMS-system that monitors the amount of SMS' sent to a woman versus the amount of SMS' delivered to the phone.

Ethics and dissemination

**Research ethics approval**	**24**	**Plans for seeking research ethics committee/institutional review board (REC/IRB) approval**<br><br>Ethical clearance has been obtained from the National Institute for Medical Research in Tanzania and written informed consent is collected for all participants. The study has been approved by ClinicalTrials.gov: NCT02509702.
**Protocol amendments**	**25**	**Plans for communicating important protocol modifications (eg, changes to eligibility criteria, outcomes, analyses) to relevant parties (eg, investigators, REC/IRBs, trial participants, trial registries, journals, regulators)**<br><br>Important study modifications will be reported to the scientific ethical committee of Denmark as well as to Clinical Trials and the National Institute for Medical Research in Tanzania.
**Consent or assent**	**26a**	**Who will obtain informed consent or assent from potential trial participants or authorised surrogates, and how (see Item 32)**<br>All eligible women are introduced to the CONCEPT study by a trained nurse. The nurse introduces the study examinations, tests, contact form and questionnaire as well as goes through the consent form. The nurses obtain written consent from all participants. The consent form has been translated to Swahili.<br><br>All nurses are thoroughly educated the study and in particularly in HPV and its relationship to cervical cancer to ensure they understand the medical background. Further, community health workers informs participants of positive HPV results at their home. The community health workers are introduced to the overall study, and a protocol has been developed for how they can deliver HPV-positive results to laypersons in an ethical appropriate and consistent manner as well as address any concerns the women may have in relation to their result.
	**26b**	**Additional consent provisions for collection and use of participant data and biological specimens in ancillary studies, if applicable**<br><br>Not relevant

**Confidentiality**	**27**	**How personal information about potential and enrolled participants will be collected, shared, and maintained in order to protect confidentiality before, during, and after the trial**<br><br>All study participants are given a unique study ID when enrolled in the study. All forms, expect for contact information forms, only contain the study ID. All forms are securely stored at each study site and contact forms are stored in separate folders. HPV samples can are only identified through the study ID in order to maintain confidentiality. Once the sample has been tested in the laboratory it is destroyed.
**Declaration of interests**	**28**	**Financial and other competing interests for principal investigators for the overall trial and each study site**<br><br>There are no competing interests in the study
**Access to data**	**29**	**Statement of who will have access to the final trial dataset, and disclosure of contractual agreements that limit such access for investigators**<br><br>Only the primary investigator and data manager from the University of Southern Denmark have access to the data. All investigators from the CONCEPT study can be granted access to the full trial dataset.
**Ancillary and post-trial care**	**30**	**Provisions, if any, for ancillary and post-trial care, and for compensation to those who suffer harm from trial participation**<br><br>No compensations.

**Dissemination policy**	**31a**	**Plans for investigators and sponsor to communicate trial results to participants, healthcare professionals, the public, and other relevant groups (eg, via publication, reporting in results databases, or other data sharing arrangements), including any publication restrictions**<br>The research findings will be communicated via a project website, scientific paper in peer-reviewed journals and popular articles. Further, to get maximum impact of the findings, a stakeholder workshop focusing on cervical cancer screening and continuity of care will be held at the beginning and at the end of the project. The invitees will include Women's groups representatives, civil society, health care providers, health sector officials and researchers. The workshop will involve stakeholders in the formulation of intervention strategies, which will enhance continuity of care among women who are screened positive. Further, at the end of the project period a policy brief workshop facilitated by a communication expert will be held and will result in the formulation of a policy brief that summarises the research findings and provides recommendations to decision makers. The policy brief will be produced and shared at a national dissemination seminar where health policy makers, Danida representatives and other relevant stakeholder are invited.<br><br>The study results will be published in peer-reviewed journals. Further, the findings will be presented at international conferences. The investigators oblige themselves to publish both positive and negative findings.
	**31b**	**Authorship eligibility guidelines and any intended use of professional writers**<br>Authorship follows the Vancouver guidelines. No use of professional writers.
	**31c**	**Plans, if any, for granting public access to the full protocol, participant-level dataset, and statistical code**<br>It is possible that the public can be granted access to the full protocol, dataset and statistical code.
**Appendices**		
**Informed consent materials**	**32**	**Model consent form and other related documentation given to participants and authorised surrogates**<br><br>Appendix A: Eligibility and Informed Consent Form<br>Appendix B: Contact Information Form
**Biological specimens**	**33**	**Plans for collection, laboratory evaluation, and storage of biological specimens for genetic or molecular analysis in the current trial and for future use in ancillary studies, if applicable**<br>No biological agents.

APPENDIX A

ConcepT

Comprehensive Cervical Cancer Prevention in Tanzania

ELIGIBILITY AND INFORMED CONSENT

Eligibility

1. Does the woman consent to be informed about the study **Y/N**
   If no, stop the recruitment process
2. Is the woman aged between 25 – 60 years of age **Y/N**
3. Is the woman pregnant? **Y/N**
4. Has the woman underwent hysterectomy **Y/N**
5. Has the woman ever been diagnosed with cervical precancer within the last 12 months? **Y/N**
6. Has the woman previously been diagnose with cervical cancer? **Y/N**
7. Is the woman menstruating now? **Y/N**

For the woman to be eligible, Question 1 – 2 should be Yes; and question 3 – 7 should be No. If eligible, read the statement of informed consent.

Statement of the Informed Consent
Research Description: You are invited to participate in this research aimed to find the appropriate means for prevention of cancer. Cancer of the cervix is the most common cancer among young and middle-aged women in our country. It develops mainly due to infection with viruses, called Human Papilloma Virus (HPV). This virus causes changes of the superficial cells of the cervix which in the long run may turn out to be cancer.

Procedures: The procedures which you will undergo entail no potential risk to you or your confidentiality. The procedure will be monitored by the study team.

Benefit: Cervical screening will identify if you have neoplastic or preneoplastic lesions. Thus, you will benefit from the dedicated standard local medical care including cervical pre-cancer lesions treatment. There will also be extra incentives such as free access to the study medical staff if you have questions during your visit.

Confidentiality: To ensure confidentiality, all questionnaires, laboratory slips and specimen will be labeled with the study ID numbers; your name will not be used.

Compensation: There will be no compensation for participation in the study

APPENDIX B

ConcepT

___________ Study number

Comprehensive Cervical Cancer Prevention in Tanzania

CONTACT INFORMATION

Facility name …………………. Date ……………………

Name: First………………………… Middle …………………. Last ……………………

Popular Name: …………………………………………….

Date of Birth: ………………………….. (Age) …………..

Residential Address:

Region: ……………………………………………….

District: ……………………………………………….

Ward: ……………………………………………….

Village: ……………………………………………….

Street name: …………………………………………..

House no.: ………………………………………….

Name of street leader/ten cell leader/popular person ………………………………

Popular landmark or person near the house: ……………………………………………….

Mobile Phone numbers: (1) ……………………….. (2) …………………………
Primary Secondary

Name and Mobile phone number of close relative or friend:

…………………………………….	…………………	…………………………
Name	Relation	Phone number

Voluntary participation: Your participation in the study is entirely voluntary; you are free to refuse to participate or to refrain from answering some questions if you do not want to. Your routine health care from the health services will not be affected in any way.

A doctor/health worker explained to me in detail about the study on detection of Human Papilloma Virus (HPV), which causes cervical cancer in women. In addition, I have been told that presence of Human Immunodeficiency Virus (HIV) increases the risk of developing cervical cancer in the presence of HPV. I understand the following procedure will be performed to detect the presence or absence of Human papilloma Virus and pre-cancerous lesions:

1. Participating in a questionnaire
2. Taking blood for HIV testing
3. Looking at the surface of cervix after insertion of speculum in the vagina
4. Taking a cervical swab using a sterile swab to detect the presence of Human Papilloma Virus
5. You may need to attend follow-up appointments at the clinic at 14 months and 28 months.

Do you consent to participate in the study and be tested for HIV? *(tic 1 box)*

- Yes, I want to participate in the study and test for HIV	☐	*Continue procedure → sign next page*
- Yes, I want to participate in the study, but I do not want to be tested for HIV because I already know that I am HIV positive	☐	
- No , I do not want to participate in the study because I do not want to be tested for HIV<br>Reason____________________________	☐	*Stop procedure → refusal book*
- No , I do not want to participate in the study for other reasons<br>Reason____________________________	☐	

For more information or in case of any problem, please contact the following:

Dr Julius Mwaiselage,<br>Director of Cancer Prevention Services,<br>Ocean Road Cancer Institute,<br>Barack Obama Road,<br>P.O. Box 3295, Dar es Salaam, Tanzania<br>Tel: 022 2127597; Fax: 022 2118704	Prof. Rachel Manongi<br>Head, Department of Community Medicine<br>Kilimanjaro Christian Medical Centre<br>Longuob Street<br>P.O. Box 3010, Moshi, Tanzania<br>Tel: 027 2754377; Fax: 027 2754381	Secretariat,<br>National Health Research Ethics Review Committee<br>National Institute for Medical Research<br>2448 Ocean Road<br>P.O. Box 9653 Dar es Salaam Tanzania<br>Tel: +255 22 2121400; Fax: +255 22 2121360

I understand the information thus obtained will be kept confidential and will be used to assist in the fight against cervical cancer in Tanzania. I understand that these procedures are generally harmless, but may occasionally cause some discomfort. I understand that I will be treated by medicines or surgery or referred to specialized centre in the event of any abnormality being detected.

I hereby express my willingness to participate in this study and to undergo the above procedures.

Thumb print

Name……………………………………………………………………………………….

Signature……………………………… Date………………………………………….

Study number………………………

Facility name ……………………...

Date ………………………………..

Health provider initials …………..

REFERENCES

1. Stewart W and Christopher PW. World Cancer Report 2014. 2014.
2. Dartell M, Rasch V, Kahesa C, Mwaiselage J, Ngoma T, Junge J, et al. Human papillomavirus prevalence and type distribution in 3603 HIV-positive and HIV-negative women in the general population of Tanzania: the PROTECT study. Sex. Transm. Dis. [Internet]. 2012;39:201–8. Available from: http://www.ncbi.nlm.nih.gov/pubmed/22337107
3. Cancer IA for R on. M IARC Monographs. Biological Agents. Volume 100 B. A review of human Carcinogens. Lyon;
4. International Agency for Cancer Research. Globocan 2012 – Estimated Cancer Incidence, Mortality and Prevalence Worldwide in 2012. Population Fact Sheets [Internet]. 2012 [cited 2015 Oct 28]. Available from: http://globocan.iarc.fr/Pages/fact_sheets_population.aspx#
5. Plotkin M, Besana G V, Yuma S, Kim YM, Kulindwa Y, Kabole F, et al. Integrating HIV testing into cervical cancer screening in Tanzania: an analysis of routine service delivery statistics. BMC Womens Heal. [Internet]. 2014;14:120. Available from: http://www.ncbi.nlm.nih.gov/pubmed/25271025
6. Dartell MA, Rasch V, Iftner T, Kahesa C, Mwaiselage JD, Junge J, et al. Performance of visual inspection with acetic acid and human papillomavirus testing for detection of high-grade cervical lesions in HIV positive and HIV negative Tanzanian women. Int. J. Cancer. 2014;135:896–904.
7. Tanzania Commission for AIDS, Zanzibar AIDS Commission, National Bureau of Statistics, Office of Chief Government Statistician Zanzibar II. HIV/AIDS and Malaria Indicator Survey 2011–12 [Internet]. … , Tanzania. Dar es Salaam, Tanzania Tanzania …. Dar es Salaam; 2013. Available from: http://scholar.google.com/scholar?hl=en&btnG=Search&q=intitle:HIV/AIDS+and+Malaria+Indicator+Survey+2011-12#0\nhttp://scholar.google.com/scholar?hl=en&btnG=Search&q=intitle:Tanzania+HIV/AIDS+and+Malaria+Indicator+Survey+2011?12#5
8. Kahesa C, Kjaer S, Mwaiselage J, Ngoma T, Tersbol B, Dartell M, et al. Determinants of acceptance of cervical cancer screening in Dar es Salaam, Tanzania. BMC Public Health [Internet]. BMC Public Health; 2012;12:1093. Available from: http://www.pubmedcentral.nih.gov/articlerender.fcgi?artid=3551792&tool=pmcentrez&rendertype=abstract
9. Lyimo FS, Beran TN. Demographic, knowledge, attitudinal, and accessibility factors associated with uptake of cervical cancer screening among women in a rural district of Tanzania: Three public policy implications. BMC Public Health [Internet]. 2012;12:22. Available from: http://www.biomedcentral.com/1471-2458/12/22
10. Kileo NM, Michael D, Neke NM, Moshiro C. Utilization of cervical cancer screening services and its associated factors among primary school teachers in Ilala Municipality, Dar es Salaam, Tanzania. BMC Health Serv. Res. [Internet]. BMC Health Services Research; 2015;15:552. Available from: http://ovidsp.ovid.com/ovidweb.cgi?T=JS&PAGE=reference&D=prem&NEWS=N&AN=26666242
11. Källander K, Tibenderana JK, Akpogheneta OJ, Strachan DL, Hill Z, Asbroek AHA Ten, et al. Mobile health (mhealth) approaches and lessons for increased performance and retention of community health workers in lowand middle-income countries: A review. J. Med. Internet Res. 2013;15:1–16.
12. Welfare M of H and S. Tanzania National eHealth Strategy June, 2013 – July, 2018. 2013.
13. Lund S, Nielsen BB, Hemed M, Boas IM, Said A, Said K, et al. Mobile phones improve antenatal care attendance in Zanzibar: a cluster randomized controlled trial. BMC Pregnancy Childbirth [Internet]. 2014;14:29. Available from:

http://www.pubmedcentral.nih.gov/articlerender.fcgi?artid=3898378&tool=pmcentrez&rendertype=abstract
14. Lester RT, Ritvo P, Mills EJ, Kariri A, Karanja S, Chung MH, et al. Effects of a mobile phone short message service on antiretroviral treatment adherence in Kenya (WelTel Kenya1): a randomised trial. Lancet [Internet]. 2010;376:1838–45. Available from: http://www.ncbi.nlm.nih.gov/pubmed/21071074
15. Pop-Eleches C, Thirumurthy H, Habyarimana JP, Zivin JG, Goldstein MP, de Walque D, et al. Mobile phone technologies improve adherence to antiretroviral treatment in a resource-limited setting: a randomized controlled trial of text mesage reminders. Aids. 2013;25:825–34.
16. Zurovac D, Sudoi RK, Akhwale WS, Ndiritu M, Hamer DH, Rowe AK, et al. The effect of mobile phone text-message reminders on Kenyan health workers' adherence to malaria treatment guidelines: a cluster randomised trial. Lancet [Internet]. Elsevier Ltd; 2011;378:795–803. Available from: http://linkinghub.elsevier.com/retrieve/pii/S0140673611607836
17. Hingle MD, Snyder AL, McKenzie NE, Thomson CA, Logan RA, Ellison EA, et al. Effects of a short messaging service-based skin cancer prevention campaign in adolescents. Am. J. Prev. Med. [Internet]. Elsevier; 2014;47:617–23. Available from: http://www.sciencedirect.com/science/article/pii/S0749379714003110
18. Chung IY, Kang E, Yom CK, Kim D, Sun Y, Hwang Y, et al. Effect of short message service as a reminder on breast self-examination in breast cancer patients: a randomized controlled trial. J. Telemed. Telecare [Internet]. 2015;21:144–50. Available from: http://www.ncbi.nlm.nih.gov/pubmed/25697492
19. Kerrison RS, Shukla H, Cunningham D, Oyebode O, Friedman E. Text-message reminders increase uptake of routine breast screening appointments: a randomised controlled trial in a hard-to-reach population. Br. J. Cancer [Internet]. Nature Publishing Group; 2015;112:1005–10. Available from: http://www.ncbi.nlm.nih.gov/pubmed/25668008
20. Odeny TA, Bailey RC, Bukusi EA, Simoni JM, Tapia KA, Yuhas K, et al. Text Messaging to Improve Attendance at Post-Operative Clinic Visits after Adult Male Circumcision for HIV Prevention: A Randomized Controlled Trial. PLoS One. 2012;7:1–7.
