# Supplementary material for: Text messages to increase attendance to follow-up cervical cancer screening appointments among HPV-positive Tanzanian women (Connected2Care): study protocol for a randomised controlled trial
Source: Trials. 2017 Nov 21;18:555. doi: 10.1186/s13063-017-2215-x (PMC5698928; doi:10.1186/s13063-017-2215-x)
Supplement: Supplementary file 2 — CONCEPT protocol. Protocol for the overall CONCEPT protocol. Trial is listed under Work Package 4. (PDF 424 kb) [file 13063_2017_2215_MOESM2_ESM.pdf]

Appendix A: Project Description

Comprehensive Cervical Cancer Prevention in Tanzania (CONCEPT)

1. Project Summary

The natural history of Human papillomavirus (HPV) in sub-Saharan Africa is not yet fully understood. As persistent HPV infection is a necessary factor in development of cervical cancer – a major health problem in sub-Saharan Africa - information about how HIV together with other risk factors interacts with HPV acquisitionand HPV persistence/clearance is warranted. Additionally, concern prevails about the quality of cervical cancer preventive strategies in sub-Saharan Africa. Against this background the project aims to provide a better understanding of the natural history of HPV with a view to HIV status, to measure the impact of a novel and simple screening method based on *Care*HPV testing and to assess how continuity of care among women who are tested HPV positive can be improved. The study will be linked up with the existing cervical cancer screening program at Ocean Road Cancer Institute (ORCI) and Kilimanjaro Christian Medical Centre (KCMC); and will include 4000 women who are attending screening service or HIV care and treatment. HPV acquisition and persistence/clearance patterns as well as the absolute risk of severe cervical precancerous lesions will be determined through a follow up study lasting 28 months. The test performance of *Care*HPV testing, liquid-based cytology and VIA will be described and the operating characteristics of the three screening methods will be assessed according to HIV status. Finally, the impact of a mobile phone intervention and a patient navigation model will be assessed and compared according to the proportion of HPV positive women who return for follow-up examinations. Additionally, the project will implement a research capacity building component focusing on transfer of knowledge and technology as well as training of PhD students. The activities will be carried out as five sub-studies: i) HPV acquisition pattern; ii) HPV persistence/clearance pattern; iii) Test performance of *Care*HPVtesting, liquid-based cytology and VIA; iv) Continuity of care among screened positive women and v) research capacity building as well as transfer of knowledge and technology

2. Objectives

With the overall aim of substantially improving cervical cancer prevention in Tanzania and thereby reduce mortality, the CONCEPT project focuses onthe natural history of human papillomavirus (HPV), which is the most important risk factor for cervical cancer.

HPV can take the form of being either a transient infection, that relatively rapidly is cleared by the host immune system or it can becomea persistent infection that may progress to high-grade cervical lesions or cervical cancer**(Fig. 1)**.In spite of the fact that cervical cancer is the most common malignancy among women in Sub-Saharan Africa (1), little is known about the distribution of HPV types, risk factors of incidence and patterns of persistence for different HPV types. The proposed project will address this lack of knowledge and provide new information about the natural history of HPV and consequences of HPV infection among HIV positive and HIV negative women.In addition, there is concern about the performance of the screening approaches applied in many sub-Saharan African countries and information on the quality of novel, affordable screening approaches that will perform well in remote areas iswarranted. Finally, in many sub-Saharan African settingsworries prevail about lack of continuity of care among women who are diagnosed with precancerous lesionsand therefor relevant treatment is not always offered. The proposed project will address these shortcomings in cervical cancer prevention. The researchwill build on the resultspreviously obtained by our research team(2-6) and will be innovative in the way that it through a comprehensive approach will use thenatural history of HPV to identify opportunities to strengthening and further develop the existing screening program in Tanzania, with a particular view to the importance of HIV positivity. In addition, with the aim to bridge the gap in continuity of care among women diagnosed with precancerous lesions,the project will develop and test a reliable and sustainable communication system through a multidisciplinary approach, involving community education, social mobilization and use of cell-phones. Thus the proposed study has the potential to produce results with immediate public health impact.

The specific objectives of the project are:

1. To assess acquisition patterns and incidence of HPV infection with a specific focus on differences in HPV acquisition, distribution of HPV types and risk factors among HIV positive and HIV negative women

2. To assess persistence of high-risk HPV infection with a specific focus on whether there are differences in HPV persistence in relation to specific HPV types and differences in risk factors for HPV persistence among HIV positive and HIV negative women. In addition, it is the aim to examine the absolute risk of HSIL or worse in relation to both one time HPV positivity and HPV persistence while taking HIV status into account

3. To evaluate the performance ofSelf collected *Care*HPV testing, health provider collected HPV test, Pap smear (liquid-based cytology) and visual inspection with acetic acid (VIA) for detection of cervical precancerous lesions with a special view to test performance among HIV positive and HIV negative women.

4. To evaluate and compare two different interventions aiming at ensuring continuity of care among women who are tested HPV positive in terms of effect and costs, and to describe barriers for not adhering to the scheduled follow-up

5. To enhance research capacity and transfer of knowledge and technologythrough the training of PhD students and the involvement of a post-doctoral fellow

3. Project's methodology

Based on the experience and success of our previous study of HPV prevalence in Tanzanian women, the present study will be linked up with the existing cervical cancer screening program in Dar es Salaam and Kilimanjaro Region. To make sure that we will include enough HIV positive women into the study, we will oversample HIV positive women. This will be done through enrolment of women attending HIV care and treatment in the two study areas.A total of 4000 women will be enrolled at baseline in the study -3500 women from the two screening settings and 500 HIV positive women from two HIV care and treatment clinics. Based on our previous experience, amongthe 3500 women recruited from the screening settings, around 700 will be high-risk (HR)-HPV positive and nearly 350 women will be HIV positive(2). Among the 500 HIV positive women recruited from the HIV clinics, an estimated 250 women will beHPV positive(2). Hence, the total study sample of 4000 women will include an estimated 950 HPV positive and 850 HIV positive women.A power calculation was based on McNemars test in the situation where we compare two diagnostic tests (1: standard test (VIA)vs 2:new test (*Care*HPV)) where the outcome is sensitivity S_1 and S_2, respectively.Based on our previous studies in Tanzania (4) it is estimated that 180-200 women will have precancerous lesions at baseline (~true positive), and we assume a significance level of 5% and that the sensitivity of the standard test is S_1=30%. We will then with 80% power be able to detect a significant difference if the sensitivity of the new test is at least S_2=44%. As we anticipate *Care*HPV testing to have a much higher sensitivity, we will have sufficient power in the present study

An overview of the study design is outlined in **Fig. 2.** In principle the study comprises a baseline visit and 2 follow-up visits:

At ***baseline*** we will collect on all participating women a cervical sample for *Care*HPV testing, a novel and simple quick test for detection of HPV.We will also obtaina liquid-based cervical swab (ThinPrep) for cytology examination (subsequently prepared and diagnosedin Denmark), high-risk Hybrid Capture (HC2) testing (in Germany), and genotyping (PCR-based HPV testing in Germany). Following this, VIA will be done with 5% acetic acid. The Tanzanian staff will receive training (re-training) in sample collection and VIA before start of the study by national trainers according to IARC guidelines. In case of a positive cytology (HSIL or worse) the woman will be called in and will be treated according to the cervical cancer screening standard of care methods in Tanzania. Similarly, all VIA positive women will be treated according to the cervical cancer screening standard of care methods in Tanzania.Information on socio-economic characteristics, lifestyle factors and reproductive history will be recorded through a personal

interview, and blood samples for HIV testing will be obtained. Before the initiation of the study, the staff in Tanzania will receive training in *Care*HPV testing.The *Care*HPV (including currently known HR-HPV types 16, 18, 31, 33, 35, 39, 45, 51, 52, 56, 58, 59, 66, and 68) will be tested within 14 days in Tanzania. At the ***first follow-up,*** taking place 14 months after inclusion,a randomly selected sample of 500 women will be trained on self-collection of a cervical swab for HPV testing. Thereafter another cervical swab (ThinPrep) will be collected (on all study subjects in the 1st follow-up) by a trained health provider. At the ***second follow-up,*** taking place 26 months after inclusion, a cervical swab (ThinPrep) will be obtained from each woman. Women who do not return to the clinic for first and second follow-up will be traced and visited at home and invited to attend the clinic for screening. If they do not wish to re-attend, they will be offered screening through a self-collected HPV sample.We anticipate a response rate in the 1st follow-up at 90% and a response rate in the 2nd follow-up at 80%. The anticipated follow-up rate is based on experiencesfrom previous follow-up studies in Tanzania(7).
The study is grouped in five work package according to the specific objectives:

Work package 1, Acquisition patterns of high-risk (HR) human papillomavirus (HPV) with a special view to HIV status: Among women testing HR-HPV negative at baseline (Fig. 2), all women testing HIV positive (~190 from the screening clinic and 250 from HIV clinics = 440 in total) as well a random sample of 560 HPV negative/HIV negative women will be invited to a new gynecological examination after 14 months (1st follow-up visit), where a health provider cervical swab (ThinPrep) sample on all women is taken.These sampleswill all be tested for HPV using HC2 and genotyping will be performed on the HPV positive samples. At the 2nd follow-up visit (at 26 months) a health provider taken cervical swab (ThinPrep) will be obtained. All samples from the 2nd follow-up will be sent to Denmark for establishment of a cytology diagnosis and the remaining material will be sent to Germany for HC2 testing and subsequent genotyping of the HPV positives.All cytology results and HPV results will be sent back to Tanzania. In case of a positive cytology (HSIL or worse) the woman will be called in and will be treated according to the cervical cancer screening standard of care methods in Tanzania.
Based on the literature (8) we estimate an HPV acquisition rate among the initially HPV negative women at 1st follow-up of around 10%, similarly an acquisition rare of 10% from the 1st to the 2nd follow-up. The association between HR-HPV acquisition and the women's age, life style characteristics, reproductive history and HIV status will be assessed.

Work package 2, Persistence patterns of high-risk HPV types and absolute risk of severe cervical lesions focusing on the importance of HIV status: Persistence/clearance patterns of HR-HPV will be determined among all women who are HR-HPV positive at baseline(Fig. 2) (~700 from the screening clinic and 250 from the HIV clinic i.e. ~950 in total). They will be invited for follow-up examinations and tested for HR-HPV after 14 and 26 months (1st and 2nd follow-up visit). For this group of women also a self-collected cervical swab will be obtained at the 1st follow-up in 500 randomly selected women. HPV genotyping will be performed among women who are HR-HPV positive during follow-up.Women who are persistently HPV positive for the same HPV type at enrolment and at 1st follow-up will be called in for a cytological examination at the respective clinic.Also in case of a positive cytology (HSIL or worse), the women will be called in for further follow-up. HR-HPV persistence/clearance patterns will be described in relation to the women's age, life style characteristics, reproductive history, HIV status and HPV genotype. We estimate a persistence rate of 40% after 14 months and of 20% after 26 months (9). Due to the fact that a cytology diagnosis is obtained on all women at the 2nd follow-up, we will also be able to look at the absolute risk of HSIL or worse in relation to both one time HPV positivity, HPV persistence and according to HIV status.

Work package 3, Test performance of *Care*HPV testing, pap smear and VIA for detection of cervical precancerous lesions: As described above, all 4000 women enrolled will be screened by use of *Care*HPV testing, Pap smear (liquid based cytology i.e. ThinPrep) and VIA at the study baseline, and the sensitivity and specificity of *Care*HPV testing and VIA will be estimated and the positive and negative predictive values calculated. The operating

characteristics of the two screening methods will be assessed according to HIV status. All VIA positive women will subsequently be treated in agreement with thecervical cancer screening standard of care methods in Tanzania. In case of a positive cytology that was not already identified through a positive VIA, the women will be called in for further follow-up.High-quality cervical liquid-based cytology and where possible also cervical biopsies will be used as Gold Standard. Based on the samples collected at the 1st follow-up, the HPV results from the self-collected brush and the health provider collected brush will be compared.

Work package 4, Continuity of care among women who are tested are HPV positive – a comparison of two different interventions: Women who are tested HPV positive at enrolment will be randomized to either a patient navigation model or a cell phone model consisting of automated SMS messages. *Patient navigation model*: A trained community health worker will be identified as the woman's patient navigator. There will be established a one-to-one relationship between the patient navigator and the woman to address anticipated barriers such as communication difficulties and difficulties with arranging transportation. *Cell phone model*: HPV positive women will receive automatically generated SMS messages, which will convey HPV result, send appointment reminders and health information during the first 12-14 months follow-up period.After 20 months, the continuity of care, based on the number of HPV positive women who return for the 1st follow-up examination after 14 months, will be compared. Additionally, the average time spent providing navigation from an HPV positive result is established to 12-14 months after and the associated cost will be calculated. Likewise the price of establishing and maintaining the system generating the SMS reminders will be measured. The differences in total costs and re-attendance between patient navigation and SMS reminders will be calculated. Re-attendance will be defined as HPV positive woman who re-attend within 12-14 months after the HPV positive result is established. Finally, HPV positive women who do not re-attend for screening after 12-14 months will be traced and interviewed. A mixed method approach, relying on structured questionnaires, in-depth interview and key informant interviews will be used to describe perceived barriers for attending 12-14 months follow-up.

Work package 5, Health service capacity building for cervical cancer prevention:Health service capacity building will be performed at primary, secondary and tertiary level. At the primary and secondary levels, key barriers for optimal use of existing communication paths for ensuring continuity of care among women diagnosed with precancerous lesions will be identified through a register based desk study. Based on the results, interview guides will be developed for in-depth interviews with health providers working at primary and secondary level and community representatives. The experiences from this assessment will be used to develop a training program in cervical cancer prevention and patient navigation that will include staff at primary and secondary health units together with community health workers in Dar es Salaam and Kilimanjaro Region. The trained community health worker will be employed as patient navigators. At tertiary level, the project will respond to the research needs set forth by ORCI and KCMC. Three PhD courses, one in Clinical Trials, one in Prevention and Control of Gynaecological Cancers and one in Policy Brief Writing will be offered at KCMC in alignment with the exiting BSU initiatives at KCMC. The PhD courses will be co-developed and co-taught by Tanzanian and Danish researchers. At both ORCI and KCMC there is a need to strengthen the capacities of researchers to undertake in-country PhD training at an international level. To address this need, four PhD studies, three Tanzanian and one Danish (co funded by SDU), will be included in the project. The Tanzanian PhD students will be recruited through public announcement of the scholarships and competitive applications.Two of the Tanzanian PhD students will be enrolled at KCMC and one at ORCI. Theywill additionally conduct 3 months of academic work each year in Denmark. The project will be performed as a twinning arrangement where the Tanzanian and the Danish PhD students will work closely together. To increase the expertise within HPV epidemiology and HPV testing in Tanzania and thereby increase the sustainability of the project outcomes, a post-doctoral fellow, Crispin Kahesa(CK), who has obtained his PhD as part of our previous research (2-6, 9) and who is presently acting as national trainer for the cervical cancer

prevention program in Tanzania, will be employed in the project. He will be visiting Institute of Medical Virology at Tuebingen University in Germany to get detailed knowledge about HPV testing and HPV analyses. In addition, to enhance local expertise in cervical cancer screening and HPV testing, a faculty exchange to the International Agency for Research on Cancer (IARC) in Lyon will also be arranged. Finally, to further upgrade CK's academic skills he will be involved as a co-supervisor of the two PhD projects focusing on Cervical Cancer Screening and Continuity of Care and write two independent papers based on the research findings.

Ethical research permissions will be obtained from the Ministry of Health in Tanzania and from local authorities in Dar es Salaam and Kilimanjaro. Ethical clearance will be obtained from the Ethical Review Board at KCMC and ORCI as well as from the Ethical Review Board of the National Institute of Medical Research. The project will follow the international ethical guidelines developed by CIOMS (Council for International Organization of Medical Sciences), placing particularemphasis on ensuring participant safety. Hence, women who have a positive cytology (HSIL or worse) will be called in and treated according to Tanzanian standard of care. Similarly, women who are persistently HPV positive will be traced, and liquid-based cytology samples will be obtained and analyzed. In case of a positive cytology result (HSILor worse), the women will be offered colposcopy directed biopsies and treatment according to the cervical cancer screening national guidelines. Informed written consent will be obtained from research participants and confidentiality guaranteed.The trial will be registered at ClinicalTrials.gov and trial analyses and reports will be made in accordance with CONSORT requirements. It is an important part of the study that all women will have a cytology examination when they exit the study after 26 months and we will make sure that all women are cared for in the best possible way.

4. Expected outputs and outcomes

The project will produce 4 PhD theses, at least 14 scientific papers published in international, peer-reviewed journals, at least 5 articles published in popular journals/magazines, at least 6 conference papers (4 national and 2 international), and a minimum of 12 research updates and policy briefs.

The expected outcomes of the project are:

- New knowledge about the natural history of HPV infection and consequences of HPV infection among HIV positive and HIV negative women
- New approaches in performing cervical cancer screening. On the basis of the research, possibleimprovements of the screening program will be identified, with a particular viewto implementation of HPV testing and improved continuity of care.
- A cadre of health staff and community health workers who are trained in cervical cancer control and prevention and who through an improved communication line will help facilitate on-going care and treatment to women who are screened positive
- Improved capacities among researchers to conduct interdisciplinary and internationally informed research on primary and secondary prevention of cervical cancer
- Decreased mortality from cervical cancer due to detection of precancerous lesions and earlier detection of cervical cancer
- Reduced poverty through enhancement of women's sexual and reproductive health. To a high degree cervical cancer is diagnosed in women at reproductive age and is thus leading to high numbers of premature deaths with substantial social and economic consequences at an individual level and in society. Prevention of cervical cancer will therefore have an impact on reduction of poverty and sustainable development in society.

5. Relevance

In Tanzania, cervical cancer is the commonest type of cancer in women, and that with the highest mortality rate. Annually, approximately 7300 new cases are diagnosed and about 4200women die from cervical cancer(11). Thus cervical cancer is a public health problem that has enormous social and economic population impact as it often affects women at reproductive

age(12). To address this public health problem, the project will provide new and important information on the natural history of HPV and how it relates to HIV, information that will be of help in the development of more efficient vaccines and screening strategies that targets HIV positive and HIV negative women. The project also aims to evaluate *Care*HPV testing (a simple to do test) as a tool for cervical cancer screening and to examine how continuity of care of women who are screened positive can be improved by two simple low cost interventions. The information generated from the project will help inform the future screening strategy in Tanzania as well as in other countries with high HIV prevalence. We will make it a priority that beside the publication of our results in scientific journals, we will communicate the results to decision makers, stakeholders and to the general population.The focus areas of our research, HPV, HIV and cervical cancer are well in line with the "Tanzanian National Health Research Policy" from 2013 that lists Communicable diseases, Non-communicable diseases and Reproductive health as top-three priority areas. Our research areas are also aligned with the Tanzanian Health Sector Strategic Plan III (HSSP III 2009-2015), supported by Danida, which also lists reproductive health and communicable and non-communicable diseases as priority areas. The project does also have relevance in relation to overall objectives and priorities of Danish development cooperation as expressed in The Right to a Better Life which states that "Denmark will be at the forefront of international efforts to promote sexual and reproductive health and rights, and in the fight against HIV/AIDS". Finally, through the linkage with KCMC, the activities are also in line with Danida priorities for research capacity building, since KCMC is one of the core institutions in the Building Stronger University (BSU) program.

6. Project plan

Since the project plan is complex and dynamic with interactions between multiple WPs, the key priorities will be to facilitate the necessary interactions between the WPs and to maintain the overall scientific direction over the life of the project. The project will start in January 2015 and end in December 2019. The existing ORCI and KCMC institutional bank accounts will be used for the project; and the existing financial and procurement regulations will be used for implementing the project in Tanzania. The main project milestones and timetable are shown on **Fig. 3**. The detailed budget is attached in the main application and it shows how the resource allocation in the project will be distributed between the institutions involved over the project period.

7. Participants, organization and management

Julius Mwaiselage is a medical doctor and epidemiologist and public health specialist. He is the Director of Cancer Prevention Services at ORCI. He has 9 years experiences in cancer epidemiological research in Tanzania and has been involved in various collaborative research projects focusing on HPV prevalence, cervical cancer screening and HPV vaccine introduction in Tanzania. He has authored more than 20 scientific papers in peer-reviewed journals and supervised 3 PhD students and more than 15 master's students. **Vibeke Rasch** is a gynaecologist and professor in global reproductive health. She has almost20 years experiences in reproductive health research in Tanzania, has acted as principal responsible party in an enhancing research capacity building project in Vietnam and has been member of the BSU steering committee. She has authored more than 100 scientific publications and supervised 16 PhD students, more than half from sub-Saharan Africa and Asia. **Susanne Krüger Kjær** is a medical doctor and professor of cancer epidemiology. Her main research areas are HPV and cancer and the importance of gene-environment interactions in cancer development with a focus on clinical utility and the translational aspect. She has authored more than 350 scientific papers, and she is the Danish primary investigator on the clinical, randomised trials of the efficacy of HPV vaccination. She has supervised more than 25 Phd students. **TwalibNgoma** is a professor of oncology. He has more than 25 years of experience in teaching, clinical work and research related to cancer. He is the executive director of ORCI and the head of clinical oncology department of Muhimbili University College of Health and Allied Science. **RachelManongi** is Head of Community Medicine Department at KCMC, she has a record of community based research with a special focus on HIV prevention and sustainable health promotion and has also been active in the establishment of a cervical cancer registry at KCMC. She has authored more than 40 papers in international journals.

The project builds on and extends existing collaboration between these Tanzanian and Danish researchers. The involved Danish researchers have solid experience in research capacity building in Tanzania and in HPV research and both have strong publication records. The proposed project will be undertaken in close coordination with the research capacity building activities conducted within the BSU initiative. The overall responsibility for the project lies with the main Tanzanian applicant. To facilitate cross-country project management, a Steering Board will be established between Tanzanian and Danish collaborators. A project management unit(PMU) will be established at ORCI. The PMU will consist of a project secretary and an accountant and will be responsible for day-to-day activities. To monitor the activities, a web-based project management tool will be established. The web tool will include detailed updated work plans linked to the work packages so partners can track project progress. Project documents will be available on the web-site. Members of the Steering Board will meet on a regular basis to ensure a continuous progress of the study. In addition, annual workshop meetings will beheld with representatives from the partner institutions.

8. Project's international dimension

There is a great international interest in cervical cancer prevention focusing on different screening modalities, HPV testing and HPV vaccination, and it is one of the areas where substantial progress has been made in recent years and it is also one of the areas where research has the greatest translational potential. The suggested project relies heavily on collaboration between researchers in Tanzania, Denmark, Germany and France who have strong expertise in HPV epidemiology, HPV testing technology, cervical cancer screening approaches, and international health. Through this international collaboration, we will obtain a strong and valuable synergy. By means of this project there will be a great opportunity for transfer of knowledge and technology to Tanzania, which in a longer perspective may be further transferred to neighbouring sub-Saharan African countries with similar high prevalence rates of HPV and HIV.

9. New knowledge

Virtually all cases of cervical cancers and high-grade precursor lesions are caused by a persistent infection with HPV(13). Although effective prophylactic vaccines against HPV have been developed, they are still relatively expensive and logistically demanding as they currently require a 3-dose regimen. Furthermore, they do not treat already existing HPV infections and related diseases. Several African cross-sectional studies of the prevalence of HPV have been performed, including our own from Tanzania where we found an HPV prevalence of 20.1% among 3700 women(2). In the same study we found that 9.3% of the women were HIV positive. In contrast, only few prospective studies on HPV epidemiology have been conducted in Africa. Of these, some had a limited sample size (14), some did only include HIV negative women(9) or exclusively used self-sampled cervical swabs(15). In spite of the fact that cervical cancer is the most common malignancy among women in Sub-Saharan Africa(1), little is known about the distribution of HPV types, independent risk factors of incidence and patterns of persistence for different HPV types. Even though HPV16 has been found to be common in sub-Saharan African women, some studies have reported HPV52 and HPV58 to be more prevalent(2). Data on which HPV types are more likely to persist in relation to HIV status are scarce, particularly in HIV positive individuals. Results from the proposed study will add important information to our knowledge about the natural history of HPV in an HIV high-risk area and will be helpful in tailoring screening programs to match the needs of HIV positive and HIV negative women.

The conventional cytology screening (Pap smear followed by colposcopically-directed biopsies) demands costly cytology laboratories with skilled and highly experienced personnel, and multiple visits at regular intervals are needed. Consequently, the Pap smear screening is neither sufficiently developed nor sustainable in less developed countries(16). Therefore, cost effective methods such as VIA have been adopted in several countries for early detection of precancerous lesions. In 2002 cervical cancer screening based on VIA was implemented at

ORCI and the program has subsequently been scaled up to cover other regions in Tanzania. However, several studies, including our own recent study, have found VIA to have a limited sensitivity(4). In contrast, HPV screening is appealing due to a higher sensitivity and a higher negative predictive value(17). However, successful HPV screening in countries with limited resources requires a simple HPV test that will perform well also in remote areas and which is affordable. Finally, self-sampling will most likely be needed to make screening practical in some settings. . In relation to these issues, our study will contribute new knowledge which can immediately be used and implemented to the benefit of the women.

A successful programme for cervical cancer prevention requires function in its entirety. The programme should ensure high levels of screening coverage of the target population, offer high quality services, have good referral systems that guarantee patient follow-up and make certain that women receive appropriate treatment. Experiences from Tanzania mainland have documented that these preconditions are often not adhered to due to a poor functioning health system and as a consequence the screening results are often not conveyed and proper action not always taken. Currently, there is no mechanism in place in Tanzania to help women navigate between the potential barriers, which may be hindering factors for obtaining confirmatory diagnosis and adequatetreatment of cervical precancerous lesions. Mobile Health, which implies the use of mobile communication devices for health care, is seen as a complementary strategy for strengthening health systems and the use of SMS has successfully been used to remind patients to take drugs and attend appointment e.g.in Kenya(18). Similarly, members of our research team have documented the effect of SMS on antenatal care attendance and skilled delivery attendance in Tanzania(7).The Patient Navigation Model is an alternative approach to enhance continuity of care among underserved populations. The navigators can be trained community health workers or peer educators who provide support and guidance (19). Patient navigation is used as a standard of care in most of Western oncological departments and has been proven to be successful in several studies e.g: Cancer control among urban African American (20) and Breast cancer screening(21). Understanding the acceptance of these two proposed models among Tanzanian women detected to have HPV will help health care professionals to develop more effective continuum of care programs by promoting attendance to HPV screening and follow-up of disease progression.

10. Publication and dissemination strategy

The research findings will be communicated via a project website, scientific paper in peer-reviewed journals and popular articles. Further to get maximum impact of the findings, a stakeholder workshop focusing on cervical cancer screening and continuity of care will be held at the beginning and at the end of the project. The invitees will include Women's groups representatives, civil society, health care providers, health sector officials and researchers. The workshop will involve stakeholders in the formulation of intervention strategies, which will enhance continuity of care among women who are screened positive. Further, at the end of the project period a policy brief workshop facilitated by a communication expert will be held and will result in the formulation of a policy brief that summarizes the research findings and provides recommendations to decision makers. The policy brief will be produced and shared at a national dissemination seminar where health policy makers, Danida representatives and other relevant stakeholder are invited.

11. Strategy for phasing out of the project

The project will be undertaken in cooperation with Ministry of Health officials and with health system representatives. Based on the research conducted within each work package, concrete recommendations for improved cervical cancer prevention will be developed and shared with health policy makers and other stakeholders. Since the PI is Director of Cancer Preventive service in Tanzania at ORCI and a member of the Ministry of Health technical working group for cervical cancer prevention and control in Tanzania, he will ensure the recommendations of the project are sent to the respective authorities and implemented. Initially most of the technical recommendations from the project will be implemented at ORCI, KCMC and among cervical cancer screening implementing partners in Tanzania after update of the cervical cancer

screening national service delivery guidelines. Furthermore, through a phased and participatory approach, the policy recommendations for the Ministry of Health, will be discussed at the relevant level of authority in order to ensure the updated policy guidelines and ministerial circulars for cervical cancer include the project recommendations on improvement, particularly on continuity of care. The Tanzanian post-doctoral and PhD students will be in the forefront in the implementation and sustainability of the comprehensive cervical cancer prevention in Tanzania given the knowledge and skills obtained in the project; and it is anticipated that they will be employed by ORCI and KCMC or even at the Ministry of Health. The established cohort of HPV positive women will be kept and maintained at ORCI for future studies on HPV persistence and clearance patterns and women's absolute risk of subsequently developing precancerous lesions (17).

Fig. 1:Natural history of human papillomavirus (HPV) infection and cervical precancerous lesions

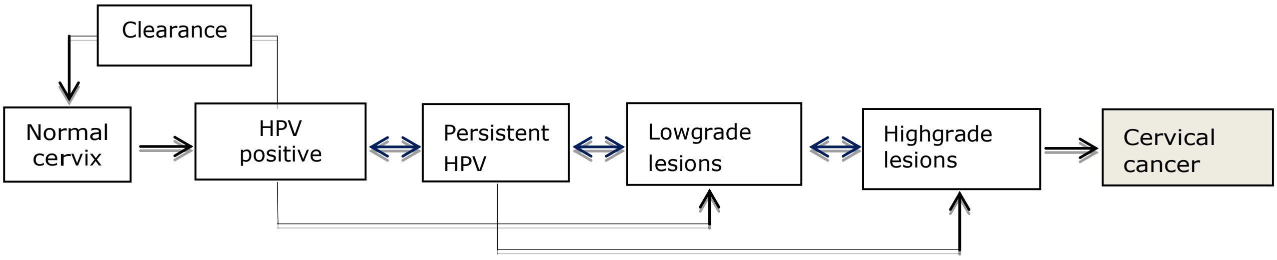

Fig 2: The schematic overview of the study design

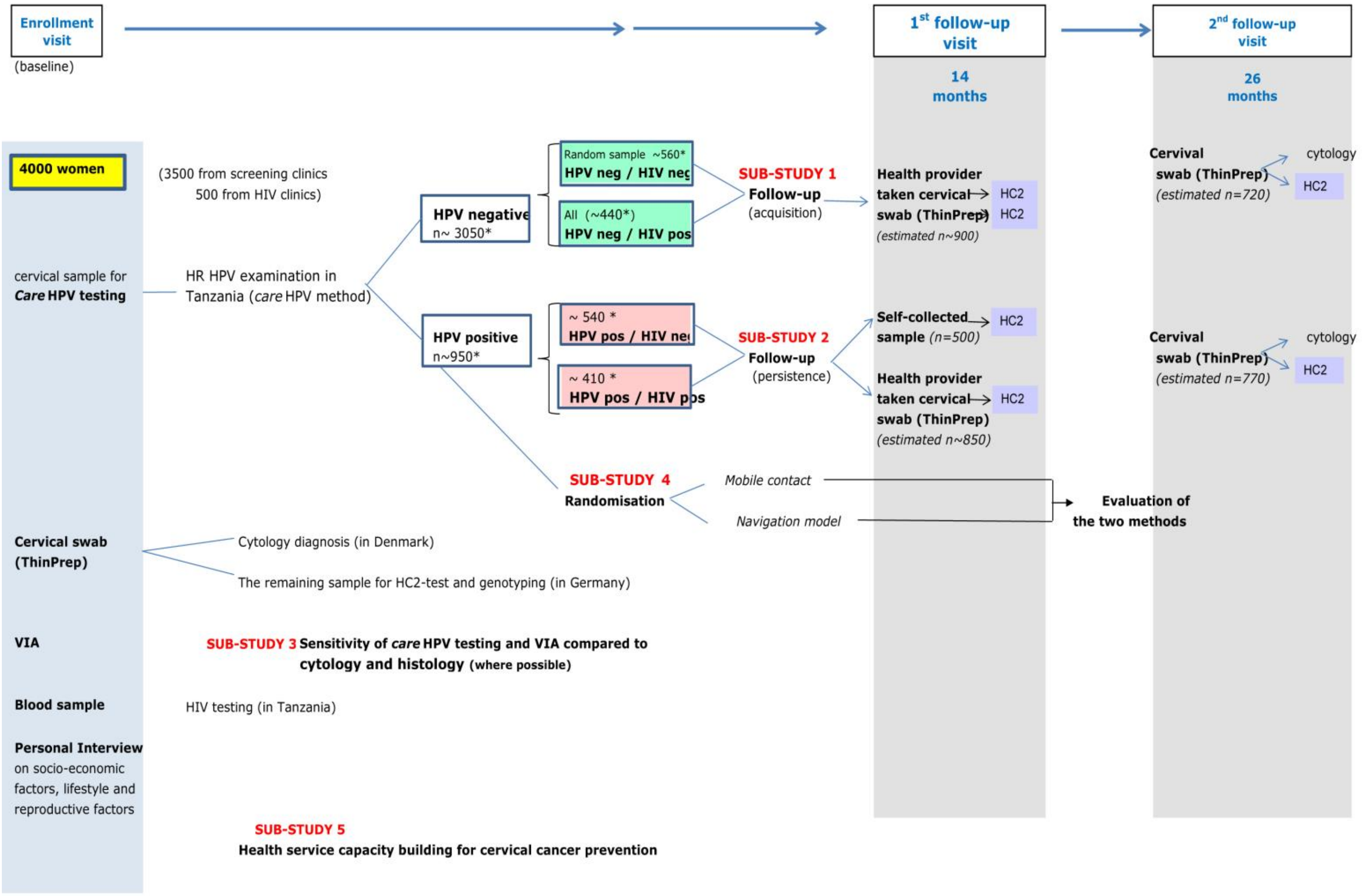

* numbers estimated on the basis of recent finding in Tanzania (Dartell et al)

Fig 3: The schematic overview of the project milestones and timetable

FIG 3: THE SCHEMATIC OVERVIEW OF THE PROJECT MILESTONES AND TIMETABLE																					
**MILESTONES**	**2015**				**2016**				**2017**				**2018**				**2019**				**Work packages**
	Q1	Q2	Q3	Q4	Q1	Q2	Q3	Q4	Q1	Q2	Q3	Q4	Q1	Q2	Q3	Q4	Q1	Q2	Q3	Q4	
Project commencement																					WP1,2,3,4,5
Announcement and recruitment of PhD students																					WP1,2,3,4
Enrollment of PhD students into universities																					WP1,2,3,4
PhD students attending university PhD program																					WP1,2,3,4
Establishment of research sites																					WP1,2,3,4
Recruitment and training of research assistants																					WP1,2,3,4
Data and specimen collection in Tanzania																					WP1,2,3
Randomization of women																					WP4
Assessment of continuity of care among randomized women																					WP4
Conducting PhD courses																					WP5
Postdoc fellow attached in research institution in France and Germany																					WP5
Publications																					WP1,2,3,4,5
PhD thesis submissions																					WP1,2,3,4
Defence of PhD thesis																					WP1,2,3,4
Dissemination of research findings																					WP1,2,3,4,5
Project completion and phasing out																					WP1,2,3,4,5

Note: Project commencement involves planning meeting of researchers, setting up of the project office, and ensuring ethical clearance is obtained from relevant bodies

Note: Project completion involves writing of the final project report, final auditing of the project, phasing out of the project to ensure sustainability

12. Main References

1.**Ferlay J**, Shin HR, Bray F, Forman D, Mathers C, Parkin DM. Estimates of worldwide burden of cancer in 2008: GLOBOCAN 2008. International Journal of Cancer 2010 Dec 15;127(12):2893-917.
2.**Dartell M**, Rasch V, Kahesa C, Mwaiselage J, Ngoma T, Junge J, et al. Human papillomavirus prevalence and type distribution in 3603 HIV-positive and HIV-negative women in the general population of Tanzania: the PROTECT study. SexuallyTransmittedDiseases. 2012 Mar;39(3):201-8.
3. **Dartell M**, Rasch V, Munk C, Kahesa C, Mwaiselage J, Iftner T, et al. Risk factors for high-risk human papillomavirus detection among HIV-negative and HIV-positive women from Tanzania. SexuallyTransmittedDiseases. 2013 Sep;40(9):737-43.
4. **Dartell MA**, Rasch V, Iftner T, Kahesa C, Mwaiselage JD, Junge J, et al. Performance of visual inspection with acetic acid and human papillomavirus testing for detection of high-grade cervical lesions in HIV positive and HIV negative Tanzanian women. International Journal of Cancer 2014 Jan 4. PubMed PMID: 24391021. Epub 2014/01/07. Eng.
5. **Kahesa C**, Kjaer S, Mwaiselage J, Ngoma T, Tersbol B, Dartell M, et al. Determinants of acceptance of cervical cancer screening in Dar es Salaam, Tanzania. BMC Public Health. 2012;12:1093.
6. **Kahesa C**, Kjaer SK, Ngoma T, Mwaiselage J, Dartell M, Iftner T, et al. Risk factors for VIA positivity and determinants of screening attendances in Dar es Salaam, Tanzania. BMC Public Health. 2012;12:1055. PubMed PMID: 23216752.
7. **Lund S**, Hemed M, Nielsen BB, Said A, Said K, Makungu MH, et al. Mobile phones as a health communication tool to improve skilled attendance at delivery in Zanzibar: a cluster-randomised controlled trial. BJOG 2012 Sep;119(10):1256-64. PubMed PMID: 22805598.
8. **Nielsen A**, Iftner T, Munk C, Kjaer SK. Acquisition of high-risk human papillomavirus infection in a population-based cohort of Danish women.Sexually Transmitted Diseases 2009 Oct;36(10):609-15.
9. **Fukuchi E**, Sawaya GF, Chirenje M, Magure T, Tuveson J, Ma Y, et al. Cervical human papillomavirus incidence and persistence in a cohort of HIV-negative women in Zimbabwe. Sexually Transmitted Diseases. 2009 May;36(5):305-11. PubMed PMID: 19295468.
10. **Olesen TB**, Iftner T, Mwaiselage J, Kahesa C, Rasch V, Ngoma T, et al. Prevalence and type distribution of human papillomavirus among 1813 men in Tanzania and the relationship to HIV status. Sexually Transmitted Diseases. 2013 Jul;40(7):592-8. PubMed PMID: 23965778. Epub 2013/08/24. eng.
11. Human Papillomavirus and Related Diseases in Tanzania. Summary Report [Internet]. ICO Information Centre on HPV and Cancer.
12. **Chokunonga E**, Borok MZ, Chirenje ZM, Nyakabau AM, Parkin DM. Trends in the incidence of cancer in the black population of Harare, Zimbabwe 1991-2010. International Journal of Cancer 2013 Aug 1;133(3):721-9. PubMed PMID: 23364833. Epub 2013/02/01. eng.
13. **Kjaer SK**, van den Brule AJ, Bock JE, Poll PA, Engholm G, Sherman ME, et al. Human papillomavirus--the most significant risk determinant of cervical intraepithelial neoplasia. International Journal of Cancer 1996 Mar 1;65(5):601-6.
14.**Veldhuijzen NJ**, Vyankandondera J, van de Wijgert JH. HIV acquisition is associated with prior high-risk human papillomavirus infection among high-risk women in Rwanda. AIDS (London, England). 2010 Sep 10;24(14):2289-92. PubMed PMID: 20613457. Epub 2010/07/09. eng.
15. **Safaeian M**, Kiddugavu M, Gravitt PE, Gange SJ, Ssekasanvu J, Murokora D, et al. Determinants of incidence and clearance of high-risk human papillomavirus infections in rural Rakai, Uganda. Cancer Epidemiology, Biomarkers &Prevention 2008 Jun;17(6):1300-7.
16. **Denny L**. Cytological screening for cervical cancer prevention. Best practice & research Clinical obstetrics & gynaecology. 2012 Apr;26(2):189-96. PubMed PMID: 22071306. Epub 2011/11/11. eng.
17. **Kjaer SK**, Frederiksen K, Munk C, Iftner T. Long-term absolute risk of cervical intraepithelial neoplasia grade 3 or worse following human papillomavirus infection: role of persistence. Journal of the National Cancer Institute. 2010 Oct 6;102(19):1478-88. PubMed PMID: 20841605.
18. **Lester RT**, Ritvo P, Mills EJ, Kariri A, Karanja S, Chung MH, et al. Effects of a mobile phone short message service on antiretroviral treatment adherence in Kenya (WelTel Kenya1): a randomised trial. Lancet. 2010 Nov 27;376(9755):1838-45. PubMed PMID: 21071074.
19. **Honeycutt S**, Green R, Ballard D, Hermstad A, Brueder A, Haardorfer R, et al. Evaluation of a patient navigation program to promote colorectal cancer screening in rural Georgia, USA. Cancer. 2013 Aug 15;119(16):3059-66. PubMed PMID: 23719894.
20. **Halbert CH**, Briggs V, Bowman M, Bryant B, Bryant DC, Delmoor E, et al. Acceptance of a community-based navigator program for cancer control among urban African Americans. Health education research. 2014 Feb;29(1):97-108. PubMed PMID: 24173501.
21. **Lee JH**, Fulp W, Wells KJ, Meade CD, Calcano E, Roetzheim R. Patient navigation and time to diagnostic resolution: results for a cluster randomized trial evaluating the efficacy of patient navigation among patients with breast cancer screening abnormalities, Tampa, FL. PloS one. 2013;8(9):e74542. PubMed PMID:
